# Development and inter-laboratory assessment of droplet digital PCR assays for multiplex quantification of 15 genetically modified soybean lines

**DOI:** 10.1038/s41598-017-09377-w

**Published:** 2017-08-17

**Authors:** Alexandra Bogožalec Košir, Bjørn Spilsberg, Arne Holst-Jensen, Jana Žel, David Dobnik

**Affiliations:** 1 Department of Biotechnology and Systems Biology, National Institute of Biology, Večna pot 111, SI-1000 Ljubljana, Slovenia; 2 Josef Stefan International Postgraduate School, Jamova 39, SI-1000 Ljubljana, Slovenia; 3 Norwegian Veterinary Institute, P.O. box 750 Sentrum, 0106 Oslo, Norway

## Abstract

Quantification of genetically modified organisms (GMOs) in food and feed products is often required for their labelling or for tolerance thresholds. Standard-curve-based simplex quantitative polymerase chain reaction (qPCR) is the prevailing technology, which is often combined with screening analysis. With the rapidly growing number of GMOs on the world market, qPCR analysis becomes laborious and expensive. Innovative cost-effective approaches are therefore urgently needed. Here, we report the development and inter-laboratory assessment of multiplex assays to quantify GMO soybean using droplet digital PCR (ddPCR). The assays were developed to facilitate testing of foods and feed for compliance with current GMO regulations in the European Union (EU). Within the EU, the threshold for labelling is 0.9% for authorised GMOs per ingredient. Furthermore, the EU has set a technical zero tolerance limit of 0.1% for certain unauthorised GMOs. The novel multiplex ddPCR assays developed target 11 GMO soybean lines that are currently authorised, and four that are tolerated, pending authorisation in the EU. Potential significant improvements in cost efficiency are demonstrated. Performance was assessed for the critical parameters, including limits of detection and quantification, and trueness, repeatability, and robustness. Inter-laboratory performance was also determined on a number of proficiency programme and real-life samples.

## Introduction

Since the early 1990s when the first genetically modified (GM) food plant was introduced, the FlavrSavr tomato, many GM plants have been more or less successfully placed on the world market^1–3^. Most countries regulate the cultivation and trade of GM organisms (GMOs)^4, 5^, and many have an authorisation system and mandatory labelling of foods that include GMOs above a certain threshold^5, 6^. In the European Union (EU), Regulation (EC) 1829/2003^7^ sets the labelling threshold for food products that contain, consist of, or are produced from authorised GMOs, at 0.9%. A consequence of this (relatively low) threshold is that distinction between authorised and unauthorised GMOs is required, and thus sensitive and specific detection and quantification methods are needed.

Most accepted methods for the detection and quantification of GMOs are DNA-based. Quantification is based on determination of the ratio between a species-specific reference gene (endogene) and a specific sequence unique to the genetic modification (the GM-event)^8^. EU legislation requires that methods for detection and quantification of both the reference gene and the specific GM-event sequence are provided by the applicant for GMO authorisation^7^. This includes the funding for validation of the method by the EU Reference Laboratory for GM Food and Feed (EURL-GMFF), assisted by the European Network of GMO Laboratories (ENGL). The validated methods are published in a database of reference methods for GMO analysis (available at: http://gmo-crl.jrc.ec.europa.eu/gmomethods/). This database also includes other formally validated GMO detection methods. The majority of the methods included in the database at present are standard-curve-based real-time PCR (qPCR) methods.

For more than a decade, GM-event–specific qPCR has been the gold standard for GMO analysis. The number of authorised GMOs has grown rapidly during this time, and separate GM-event–specific detection methods are now only exceptionally cost effective. Development and application of multiplex and screening methods can lower the costs of GMO analysis considerably, although they can also face several limitations. Several PCR^9–12^ and qPCR^13–17^ multiplex methods have been developed. One of these, a qualitative screening qPCR multiplex (5-plex) approach that detects p35S, tNOS, ctp2-cp4-*epsps*, *bar* and *pat* screening elements has also been validated^13^. These multiplex methods are predominantly qualitative. It has proven difficult to develop quantitative qPCR methods with high levels of multiplexing. To the best of our knowledge, only two duplex methods have been inter-laboratory validated for quantitative analysis; one in Europe^15^ and one in Japan^18^. The labelling threshold in the EU is not based on the individual concentration of GM-events. Instead, the labelling required is based on the total concentration of GM-events per ingredient, with an ingredient in this context interpreted as a species^8^. This opens the possibility to take a different approach, in which all GMOs from a species can be quantified simultaneously using one fluorophore^8^.

Digital PCR (dPCR) as a technique was described by Vogelstein and Kindler for the first time in the early 1990s^19^. The concept of dPCR is relatively simple. The amplification of targets in a sample is performed in hundreds, thousands, or even millions of partitions. Each partition acts as a separate qualitative test with a digital (positive or negative) outcome (hence dPCR), regardless of the amplitude of the signal. By counting the negative and positive partitions, the number of DNA copies in a sample can be calculated using the Poisson distribution^20^. This gives dPCR an advantage over qPCR, as partial PCR inhibition does not affect the quantification. Today, two dPCR technologies are available: microfluidic or chip-based digital PCR (cdPCR), and emulsion-based or droplet digital PCR (ddPCR)^20^. The applicability of a ddPCR multiplex in GMO analysis has recently been shown for duplex^21–23^, 4-plex, and 10-plex assays for GM maize^8^.

At the time of writing, there were 12 soybean GM-events authorised in the EU under regulation (EC) 1829/2003^7, 24^: 11 single GM-events and one hybrid stack (cross of two authorised single GM-events: MON87701 × MON89788) (Supplementary Table S1). In the EU, a hybrid stack is legally treated as a new GM-event, and thus requires separate authorisation. These 12 GM-events can be detected with 11 GM-event–specific PCR assays (Table 1). By 22^nd^ of July, 2016, valid European Food Safety Authority (EFSA) applications for authorisation have been submitted and are pending for an additional eight soybean GM-events. At present, these are regulated under the so-called Low Level Presence (LLP) Regulation EC 619/2011^25^. This means that the presence of each of these eight soybean GM-events is tolerated in feeds, but not in foods, at concentrations below 0.1%. Four of these eight GM-events are hybrid stacks, where the individual GM-events are authorised (305423 × 40-3-2; MON87769 × MON89788; MON87705 × MON89788; MON87708 × MON89788). These four stacks can be detected with five GM-event–specific assays that are already among the assays that detect EU-authorised single GM-events (Table 1). The remaining four GM-events that fall under LLP Regulation EC 619/2011^25^ can be detected with four GM-event–specific assays with legal status as “valid EFSA applications” (Table 1).

**Table 1:** EU authorised and tolerated soybean GM-event, together with validated qPCR methods for their detection and quantification, and specified ddPCR multiplex.

Event/species^a^	Unique identifier^b^	Legal status	Detected by	EURL-GMFF method reference^c^
MON87701	MON-877Ø1-2	Authorised	6-plex	QT-EVE-GM-010
MON87708	MON-877Ø8-9	Authorised	6-plex	QT-EVE-GM-012
MON87769	MON-87769-7	Authorised	6-plex	QT-EVE-GM-002
305423	DP-3Ø5423-1	Authorised	6-plex	QT-EVE-GM-008
BPS-CV127-9	BPS-CV127-9	Authorised	6-plex	QT-EVE-GM-011
A2704-12	ACS-GMØØ5-3	Authorised	7-plex/11-plex	QT-EVE-GM-004
MON89788	MON-89788-1	Authorised	7-plex/11-plex	QT-EVE-GM-006
MON40-3-2	MON-Ø4Ø32-6	Authorised	7-plex/11-plex	QT-EVE-GM-005
356043	DP-356Ø43-5	Authorised	7-plex/11-plex	QT-EVE-GM-009
A5547-127	ACS-GMØØ6-4	Authorised	7-plex/11-plex	QT-EVE-GM-007
MON87705	MON-877Ø5-6	Authorised	7-plex/11-plex	QT-EVE-GM-003
DAS-68416-4	DAS-68416-4	Valid EFSA application	11-plex	QT-EVE-GM-013
FG72	MST-FGØ72-2	Valid EFSA application	11-plex	QT-EVE-GM-001
DAS-44406-6	DAS-444Ø6-6	Valid EFSA application	11-plex	QT-EVE-GM-015
DAS-81419-2	DAS-81419-2	Valid EFSA application	11-plex	QT-EVE-GM-014
Soy (*Le*1)	—	—	6-plex/7-plex/11-plex	QT-TAX-GM-002

^a^Name of GMO used in the EU register of authorised GMOs^24^, except soy (*Le*1). ^b^Unique identifier according to OECD guidelines^34^. ^c^ http://gmo-crl.jrc.ec.europa.eu/gmomethods/.

The objective of the present study was to carry out an inter-laboratory comparative assessment to develop and test the performance of quantitative ddPCR multiplex assays that cover all of the present EU authorised soybean GM-events. Two quantitative ddPCR multiplex assays, a 6-plex and 7-plex, were designed, covering the 11 soybean GM-events authorised in the EU. With the addition of four tolerated GM soybean lines falling under LLP Regulation EC 619/2011^25^, the 7-plex was extended to an 11-plex assay. This introduced sufficient flexibility in the assays developed to cover all pending authorisations.

The 6-plex, 7-plex and 11-plex assays quantify the species-specific gene *Le*1 and either five (MON88701, MON87708, MON87769, 305423, BPS-CV127-9), six (A2704-12, MON89788, MON40-3-2, 356043, A5547-127, MON87705) or ten (A2704-12, MON89788, MON40-3-2, 356043, A5547-127, MON87705, DAS-68416-4, FG72, DAS-44406-6, DAS-81419-2) GM-events, respectively (Table 1). Of note, the 6-plex assay targets all five soybean GM-events that do not contain any of the five most common screening elements (p35S, tNOS, ctp2-cp4-*epsps*, *bar* and *pat*)^15^. This assay is therefore suitable for application alongside qPCR screening with, e.g., the commonly used 5-plex screening assay published by Huber *et al*.^13^.

The three multiplex ddPCR assays presented here (Table 1) are based on the combination of validated qPCRs and were designed to facilitate the testing for legal compliance in the EU. The performance with respect to sensitivity, repeatability, trueness and robustness of all three multiplexes was tested, including an inter-laboratory reproducibility study. The results and applications of the assays are discussed.

## Results and Discussion

### Selection of multiplexing approach

It is important to define the scope, performance criteria, and target values up-front for method development, validation, and application. This facilitates benchmarking and can lead to more efficient use of resources^26, 27^. The multiplex ddPCR assays reported here were developed with a clearly defined scope: fitness for detection and reliable quantification of all EU-authorised and LLP-regulated (and tolerated) soybean GM-events, in line with current EU legal requirements for GMFF^7, 25^. Quantification can be carried out per GM-event (line) or per species (ingredient). The EU labelling regulation^7^ requires labelling on the basis of the GMO concentration per ingredient. This means that it is the sum of the GM-event concentrations per ingredient that must be determined, and thus the identification or quantification of each single GM-event is not necessary. On the other hand, the LLP regulation^25^ applies only to feeds, not to foods, and LLP-regulated GMOs must be quantified individually in the EU^25^. However, these GMOs are rarely observed and their presence is tolerated up to a maximum of 0.1% (mass/mass) of an individual GMO per ingredient (species). If the presence of all EU-authorised or tolerated soybean GM-events does not exceed these thresholds in a feed product, then the product is legally compliant in the EU, except, if the product would contain other un-authorised GMO. Multiplex quantification of groups of soybean GM-events (authorised and tolerated) therefore appears to be an attractive, convenient, and potentially cost-efficient, solution.

The EURL for GMFF and the ENGL launched the first guidelines for performance assessment and acceptance of methods for legal compliance with EU GMO legislation in 2005. These guidelines have since been revised twice (2008, 2015). The latest version^27^ contains specific performance criteria and target values, and these were adopted without modifications for the present study.

### Specificity assessments

The specificity of the assays was determined *in silico*. The interactions between primers and probes were determined using the Autodimer^28^ software and the multiplex specificity was predicted using the ePCR^29^ software. No significant risk of dimers between pairs of oligonucleotides (primers/probes) was observed. In multiplex PCR, all of the possible combinations of primers can generate an unintended PCR product, if a matching template is present by chance. All of the possible primer combinations were analysed for each multiplex using 20 publically available GMO sequences (both EU authorised and unauthorised), as listed in Supplementary Table S2. In addition, the soybean and maize genomes were included. The ePCR^29^ software was used as described (see Materials and Methods). For all three multiplexes, the expected products were found, except for A2701-12, where the sequence that covers the PCR is not publically available and thus not present in the template database. ePCR identified one hypothetical unintended 207-bp amplicon that was generated with the reverse primer for MON40-3-2 alone and the SYHT0H2 EU unauthorised GM soybean line as template (unique identifier, SYN-ØØØH2-5). SYHT02 contains a 254-bp inverted repeat spanning positions 3384 to 3637; in GeneBank accession JC050419. However, this hypothetical amplicon does not match any of the probes in the multiplex soybean assays. To test this, we analysed SYHT0H2 in duplex MON40-3-2 and *Le*1 ddPCR assay. SYHT0H2 alone yielded no signal, which demonstrated that this hypothetical PCR product does not yield false-positive results. In addition, we mixed MON40-3-2 and SYHT0H2 at concentrations of 7.3% and 1.1%, respectively. This mixture was quantified to 7.9%, which corresponded to a bias of 8.2%, which demonstrated that the quantification was not affected by the presence of SYHT0H2. Classical PCR was used to explore whether it was possible to visualise the 207-bp PCR product. First a gradient PCR with annealing temperature from 50 °C to 72 °C was performed. There were no bands of the expected size, although there was a shadow at approximately 200 bp at 50 °C. The PCR was repeated with the 50 °C annealing temperature and a fast cycling programme (and the normal programme), but the predicted PCR product could not be visualised. It is possible that the template can fold back on itself and outcompete primer binding. It was concluded that this hypothetical PCR product does not interfere with the ddPCR multiplexes.

### Material characterisation and performance comparison

For pre-validation and to compare the performance of simplex, duplex (GM-event and reference gene) and multiplex (GM-events and reference gene) ddPCR assays, the absolute and relative GM-event concentrations were determined in reference materials (Table 2). The trueness of the methods were calculated as the bias compared to the certified values, and the associated method performance parameter was set to bias ≤ ±25%, as is commonly used for qPCR^26, 27^ minimum performance. The bias was <25% in all three cases. Multiplex assays were thus deemed suitable for further performance assessment. Based on this dataset (Supplementary Table S3), GM-event and endogene concentrations (copy numbers) were assigned to each individual reference material. A DNA mix of the 15 GM-events was then prepared with the copy numbers as described in Supplementary Table S4.

**Table 2:** Comparison of quantitative performance for simplex, duplex and multiplex ddPCR assays.

GM soybean line	CRM (% m/m)	Quantitative performance (%)									
		Simplex		Duplex		6-plex		7-plex		11-plex	
		GM-event	Bias (%)	GM-event	Bias (%)	GM-event	Bias (%)	GM-event	Bias (%)	GM-event	Bias (%)
MON87701	99.94	104.1	4.2	97.6	−2.3	94.3	−5.6	—	—	—	—
MON87708	99.05	105.3	6.3	101.8	2.8	101.9	2.9	—	—	—	—
MON87769	99.94	101.6	1.7	95.8	−4.1	95.3	−4.6	—	—	—	—
305423	10	8.6	−14.0	8.7	−13.0	8.2	−18.0	—	—	—	—
BPS-CV127-9	96.32*	58.9	22.2	54.7	13.5	52.8	9.5	—	—	—	—
A2704-12	99.99	102.9	2.9	93.8	−6.2	—	—	96.7	−3.3	94.8	−5.2
MON89788	100	99.7	−0.3	95.4	−4.6	—	—	89.7	−10.3	90.4	−9.6
MON40-3-2	10	10.4	4.0	9.3	−7.0	—	—	10.7	7.0	9.2	−8.0
356043	10	10.1	1.0	11.0	10.0	—	—	10.9	9.0	9.9	−1.0
A5547-127	99.99	88.4	−11.6	94.4	−5.6	—	—	93.7	−6.3	93.0	−7.0
MON87705	99.4	95.3	−4.1	99.8	0.4	—	—	98.4	−1.0	98.0	−1.4
DAS-68416-4	10	12.2	22.0	12.0	20.2	—	—	—	—	9.8	−2.0
FG72	99.99	106.3	6.3	102.8	2.8	—	—	—	—	111.1	11.1
DAS-44406-6	98.60	90.9	−7.8	117.6	19.3	—	—	—	—	110.1	10.7
DAS-81419-2	98.60	97.7	−0.9	88.1	−10.6	—	—	—	—	85.1	−13.7

CRM, Certified reference material. *CRM diluted by a factor of 2, and measured value thus compared to 48.2% (m/m), and not certified value of >96.3%.

### Estimation of the limits of detection and quantification

The lower limit of detection (LOD), the lower limit of quantification (LOQ), and the dynamic range (quantification range) were estimated using a dilution series of the mixed DNA that targeted all of the GM-events in the three multiplex assays. The observed copy numbers for the *Le*1 reference gene in all three assays and at all dilutions were compared and used as an additional control for data comparability across assays.

A total of 10 test samples (DNA mixes 1–10) were prepared from the reference materials, with the purpose of determining the absolute LOD (LOD_abs_) and LOQ (LOQ_abs_). DNA mix 1 was a mix of all 15 reference materials, and DNA mixes 2–10 were prepared as serial dilutions of DNA mix 1 in sterile de-ionised water (see Methods). The assigned copy numbers for these DNA mixes were calculated from the material characterisation data (Supplementary Table S4). For DNA mix 1, the copy number was verified by duplex ddPCR assays for each of the 15 GM soybean lines (Supplementary Table S5), and the sum of the soybean GM-event copy numbers present in the individual multiplexes was calculated and compared to the assigned copy number (Supplementary Table S6). The bias between the assigned and experimentally determined copy numbers in DNA mix 1 was well below the 25% acceptance threshold for all three assays (Supplementary Tables S5 and S6). Therefore, the assigned values for further comparisons were 26282 copies for *Le*1, and 2557, 2612 and 4658 copies of GM-events covered by 6-plex, 7-plex and 11-plex, respectively (Supplementary Table S6).

Twelve replicates were tested for the 6-plex and 11-plex ddPCR assays, and 14 replicates were tested for the 7-plex ddPCR assay, on three separate days. The LOD_abs_ was estimated to 8 copies per reaction for 6-plex, 10 copies per reaction for 7-plex, 18 copies per reaction for 11-plex, and 17 copies per reaction for *Le*1. The LOQ_abs_ was estimated as 38 copies per reaction for 6-plex, 39 copies per reaction for 7-plex and 11-plex, and 36 copies per reaction for endogene *Le*1 (Table 3 and Supplementary Tables S7–S10). The observed repeatability standard deviation RSD_r_ between replicates was consistently <25% (acceptance limit^26, 27^), with 39 or more target copies/reaction (Table 3). It is therefore reasonable to estimate that the LOQ_abs_ is 40 copies/reaction. As there were no negative replicates observed with 18 or more target copies/reaction (Table 3), it is therefore reasonable to estimate that the LOD_abs_ is 20 copies/reaction. Bias to assigned copy numbers was also determined for all three multiplexes and for *Le*1 (Supplementary Tables S7–S10). Bias was below 25% for all of the assays and DNA mixes, with the exception of the 11-plex assay for DNA mix 2, where the bias was slightly over 25% (26.11%).

**Table 3:** LOD_abs_ (bold) and LOQ_abs_ (underlined) according to total copy numbers per reaction.

DNA mix	6-plex		7-plex		11-plex		*Le*1	
	Mean copy number	RSD%	Mean copy number	RSD%	Mean copy number	RSD%	Mean copy number	RSD%
1	2229	5.4	2429	7.5	3633	7.3	22903	5.9
2	331	10.7	391	10.1	574	7.1	3382	6.0
3	189	6.8	186	9.1	316	10.1	1831	6.1
4	38	12.6	39	15.0	68	7.1	378	7.6
5	18	31.6	22	25.9	39	11.7	194	11.0
6	**8**	**26.6**	**10**	**34.1**	**18**	**36.7**	87	13.1
7	5^a^	ND^b^	4^a^	ND^b^	12	34.2	36	17.6
8	Neg.	Neg.	Neg.	Neg.	7^a^	ND	**17**	**28.5**
9	Neg.	Neg.	Neg.	Neg.	Neg.	Neg.	5^a^	ND
10	Neg.	Neg.	Neg.	Neg.	Neg.	Neg.	Neg.	Neg.

^a^At least one replicate was negative. ND, not determined due to negative replicate(s). Neg., all replicates were negative.

To estimate the relative LOD (LOD_rel_) and relative LOQ (LOQ_rel_), a second set of DNA samples (DNA mix I–VII) was prepared with an assigned constant *Le*1 copy number per reaction, as 50000, and decreasing GM-event copy numbers (Supplementary Table S11). The observed bias in the calculated GM-event percentages (GM-event copy number relative to *Le*1 copy number) was ≤25% for all of the tests that contained 100 or more GM-event copies (assigned) per reaction, except for DNA mix III with the 11-plex ddPCR assay, where the bias was 31.64% (Supplementary Table S12). The LOQ_rel_ for the 6-plex, 7-plex and 11-plex assays were 0.20%, 0.21% and 0.44%, respectively, whereas the LOD_rel_ was 0.015%, 0.017%, and 0.028%, respectively (Supplementary Table S12). Comparison of the copy numbers in LOQ_rel_ to LOQ_abs_ (39 copies) suggested that the LOQ_rel_ might be lower than that seen in Supplementary Table S12, as closer to 0.08% for all three multiplex assays. Also the LOD_abs_ determined on DNA mix VII (12 copies) suggested that the overall LOD_abs_ might be six copies lower than estimated (18 copies) for the 11-plex assay (Supplementary Table S12 and Table 3).

### Dynamic range

The dynamic range is the range of concentrations that is compliant with quantifiability and includes the LOQ. The quality criteria and acceptance values for the dynamic range include linearity (R^2^ ≥ 0.98), precision (RSD_r_ ≤ 25%) and trueness (bias ≤ 25%)^26, 27^. Furthermore, the current EU guidelines for acceptance of qPCR methods for legal compliance for GMO testing propose that the upper end of the dynamic range includes at least 2520 GM-event target copies/reaction and 56000 reference gene copies/reaction. The dynamic range should also include the relative concentration range from 0.09% to 4.5%^27^.

**Figure 1:**
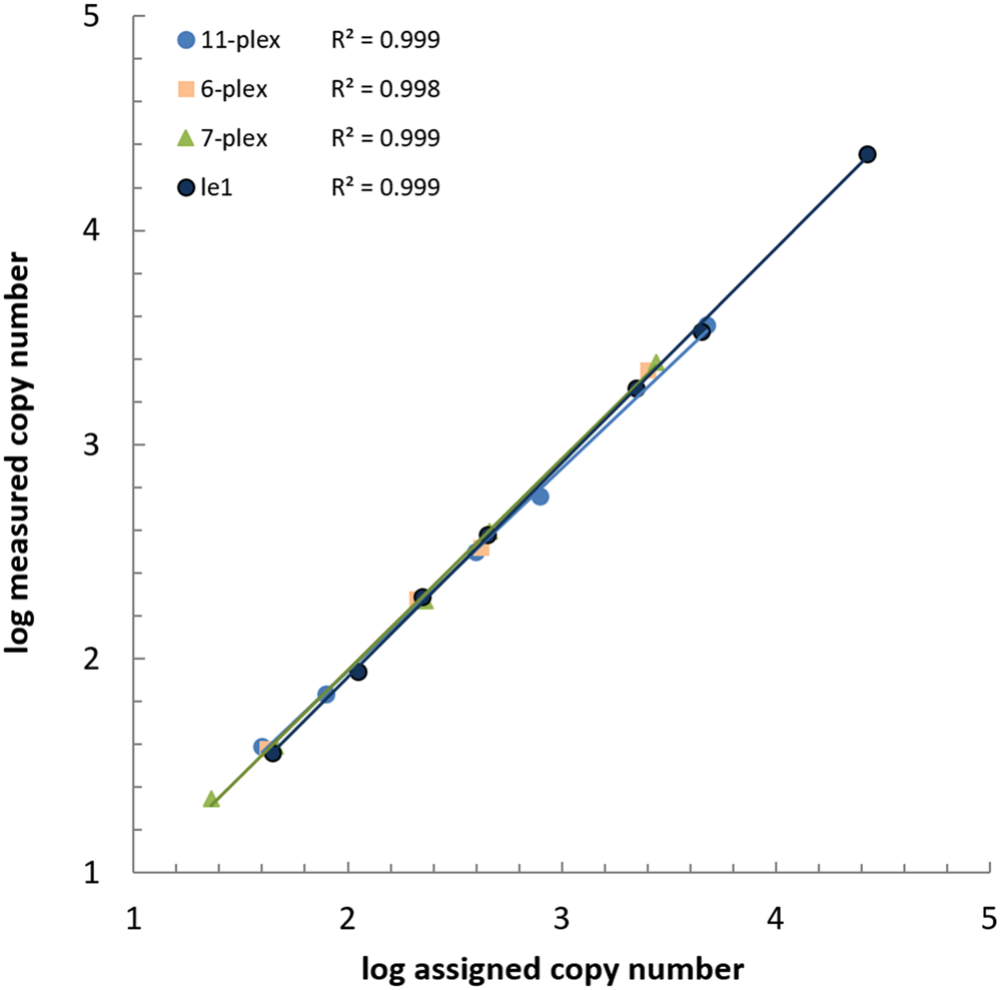
Dynamic range of the multiplex ddPCR assays. All of the assays demonstrate a very high degree of linearity (R^2^ > 0.998) for all concentrations above the estimated limit of quantification (R^2^, 0.9985 for GM-event targets in the 6-plex, 0.9995 for GM-event targets in the 7-plex, 0.9987 for GM-event targets in the 11-plex, and 0.9989 for the *Le*1 reference gene). Each data point represents the mean of three independent experiments with four replicates, except for the 7-plex, where two additional replicates were included for each data point in the third experiment.

The experimentally assigned numbers of copies of DNA mix 1 and its dilutions were plotted against the measured numbers of copies (Fig. 1) for the *Le*1 reference gene and the total GM-event content for each DNA mix. For all of the concentrations that exceeded the estimated LOQ_abs_, the coefficient of variation, R^2^, was >0.998, which indicated that the dynamic range included at least 40 to 2200 copies of the GM-event and 40 to 22000 copies of the endogene for all of the multiplex ddPCR assays. The mean observed GM-event copy numbers for the 6-plex and 7-plex assays for the least diluted samples were slightly lower than the 2520 required for qPCR, whereas the GM-event copy number for the 11-plex was well above this (Table 3). The mean observed *Le*1 reference gene copy numbers for all three assays for the least diluted sample were approximately 40% of the 56000 required for qPCR (Table 3). With further experiments where the constant *Le*1 copy number was 50000, the RSD was <25% (Supplementary Table S13), and the dynamic range was thus extended, as much closer to 56000 copies. The relative concentration range of 0.09% to 4.5% was partially satisfied by all of the assays. The lower level (LOQ_rel_) was 0.20%, 0.21% and 0.30% for 6-plex, 7-plex and 11-plex assays, respectively (Supplementary Table S12), and the upper level was well above 4.5%, at 9.61%, 10.50% and 18.07%, respectively (calculated from the data in Supplementary Tables S7–S10). Given the jump in RSD (Supplementary Table S12) additional dilutions between mix III and IV, could additionally lower the lower level of LOQ_rel_, and shift it more toward the 0.09% limit. The observed linearity (Fig. 1) and accuracy (Table 3) were acceptable for all of the tested concentrations ≥LOQ.

### Interlaboratory performance assessment

To assess the fitness for purpose and to test the inter-laboratory performance of the multiplex ddPCR assays, six real-life samples from routine testing for the presence of GMOs, and two proficiency-test samples from the US Department of Agriculture (USDA)/Grain Inspection, Packers and Stockyards Administration (GIPSA) proficiency-testing programme were selected (Supplementary Table S14). The robust means of the proficiency tests and qPCR results obtained by the National Institute of Biology (NIB, Slovenia) using GM-event–specific methods under ISO 17025 accreditation were used as accepted reference values. The results of the inter-laboratory comparison are presented in Table 4 and Supplementary Tables S15–S17.

**Table 4:** Inter-laboratory comparison of results for 6-plex and 7-plex ddPCR assays for soybean GM-event quantification.

Sample	Assigned GM-event (%)^a^	Sum GM-event (6-plex + 7-plex) (%)		Bias (%)	
		NIB	NVI	NIB	NVI
G023/11	0.91	1.03	1.06	13.2	16.5
G071/15	94.20	91.39	91.92	−3.0	−2.4
226/14	2.70	2.42	2.47	−10.4	−8.5
272/14^b^	1.29	1.40	1.39	8.5	7.8
274/14^b^	2.26	2.34	2.63	3.5	16.4
1/15	0.94	0.95	1.12	1.1	19.1
75/15	91.38	90.59	96.29	−0.9	5.4
116/15	1.68	1.73	1.91	3.0	13.7

^a^Assigned GM-event, value determined by NIB using GM-event–specific qPCR methods under ISO 17025 accreditation. ^b^USDA proficiency test sample.

The final protocols for the 6-plex and 7-plex assays were tested independently at the NIB and the Norwegian Veterinary Institute (NVI) to determine the method performance on real-life samples. This provided information on the accuracy (i.e., trueness [bias] and precision [repeatability *RSD*_*r*_ and reproducibility *RSD*_*R*_ relative standard deviation]). The overall performances were completely comparable between these two laboratories, with bias <21% (Table 4 and Supplementary Table S15). When compared in detail, all of the results fulfilled the criteria, with the exception of the repeatability between days for sample 274/14 tested at NVI (Supplementary Table S17), where RSD_r_ was slightly higher than acceptable (41.78% > 35%) for the 7-plex assay (mean GM-event target copies/reaction, 135).

The acceptance criteria were very strict and were originally developed for single GM-event–specific qPCR methods. At very low GM-event concentrations (<100 copies/reaction, or <0.09%) the EURL-GMFF/ENGL requirements are less strict (the EU guidelines for acceptance of qPCR methods accepts RSD_r_ ≤ 50%)^27^. The observed exceptions from compliance were almost consistently associated with concentrations near or below 100 copies/reaction. For sample 274/14, the RSD_r_ observed for 6-plex (46.66%) at the NVI was therefore acceptable (Supplementary Table S16). At the NIB, the repeatability was just above the acceptable value (52.48%) for 6-plex for sample 116/15; on the other hand, the RSD_r_ for the GM-event percentage was well below 25% (15.32%), and the result was considered acceptable (Supplementary Table S16). The observed reproducibility standard deviation (RSD_R_) and bias were consistently acceptable for all samples (<25%; Table 4). It is therefore reasonable to conclude that the ddPCR assays are reliable and fit for purpose.

### Accuracy (trueness and precision)

The accuracy of the 11-plex assay was independently tested at NIB, and the results of these experiments were combined with the results for the 6-plex assay from the inter-laboratory trial (Table 5 and Supplementary Table S18), using the same routine and proficiency-test samples (Supplementary Table S13). Two additional samples (49/15, G158/15) were also included (Supplementary Table S13). The bias of the measured GM-event concentration was <25% for all of the samples, except for sample 274/14, for which the observed bias was 27.9%, which was just above the threshold (Table 5). However, the RSD_r_ was acceptable for both the 6-plex and 11-plex assays (<25%) (Supplementary Table S17), although the combined data led to a bias that was slightly higher than acceptable. The observed RSD_r_ for sample 49/15, which contained only targets for the 11-plex, was just above an acceptable level (35.31% > 35%), but the bias to the assigned GM-event was <25% (Supplementary Table S18), and with LOQ_rel_ as 0.44% for the 11-plex (Supplementary Table S12), these results were acceptable.

**Table 5:** Accuracy (trueness and precision) determined by analysis of routine and proficiency-test samples at NIB with 6-plex and 11-plex ddPCR assays for quantification of GMO soybean.

Sample	Assigned GM-event (%)^a^	Sum GM-event (6-plex + 11-plex) (%)	Bias (%)
G023/11	0.91	0.97	6.6
G071/15	94.20	90.23	−4.2
G158/15	<0.10	0.07	ND^b^
226/14	2.70	2.49	−7.8
272/14^c^	1.29	1.31	1.6
274/14^c^	2.26	2.89	27.9^d^
1/15	0.94	1.03	9.6
49/15	0.48	0.53	10.4
75/15	91.38	91.88	0.5
116/15	1.68	1.92	14.3

^a^Assigned GM-event, value determined by NIB using GM-event–specific qPCR methods under ISO 17025 accreditation. ^b^Bias cannot be calculated because assigned value is below level of quantification for qPCR, and it can only be determined that there is <0.1% of GM soy present. ^c^USDA proficiency test sample. ^d^Value not compliant with acceptance values for dynamic range (quantifiability); i.e., RSD_r_ ≤25%, and bias ≤25%^26, 27^.

### Robustness

Robustness is assessed through deliberate introduction of minor modifications to the protocol, accompanied by a comparison of the results with those obtained with the original protocol. The acceptance criteria adopted here were that the modified protocol should meet the same target values as the original protocol, with accepted deviation of <25%. Robustness was assessed using three deliberate modifications: (1) use of an automated droplet generator (DG32); (2) reduced reaction volume (18 µL instead of 20 µL); and (3) use of a thermal cycler with a slower ramp rate (Table 6). The experiments were carried out with two sample concentrations (dilutions), which were intended to challenge the LOQ and LOD. The three multiplex assays demonstrated compliance with all of the performance parameters and acceptance values^27^, with a few minor exceptions observed.

**Table 6:** Robustness testing.

System	dilution	Protocol										
		original		1^a^			2^b^			3^c^		
		GM-event (%)	RSD %	GM-event (%)	RSD %	Bias %	GM-event (%)	RSD %	Bias %	GM-event (%)	RSD %	Bias %
6-plex	60×	10.54	27.52	11.26	19.55	6.88	8.15	11.29	−22.63	10.73	14.86	1.77
	240×	13.76	68.12	7.49	47.18	45.56	13.53	30.35	−1.64	12.44	23.22	−9.6
7-plex	60×	9.88	8.95	10.04	10.81	1.58	9.11	7.71	−7.79	10.62	12.48	7.47
	240×	12.25	32.64	11.41	66.83	−6.82	16.03	30.76	30.9	9.9	29.92	−19.2
11-plex	60×	20.31	7.2	22.66	37.36 ^d^	11.54	18.04	9.6	−11.2	17.65	16.36	−13.12
	240×	20.75	26.16	20.47	35.4	−1.37	18.51	27.15	−10.8	22.64	34.22	9.12

^a^Protocol 1: use of automated droplet generator (DG32). ^b^Protocol 2: reduced reaction volume (18 µL instead of 20 µL). ^c^Protocol 3: use of thermal cycler with slower ramp rate. ^d^RSD higher than acceptance value (35%) for dynamic range (quantifiability).

For modification (1), but not for (2) and (3), the 11-plex assay yielded unacceptably high RSD_r_ for the 60× dilution (Table 6). The observed copy numbers were lower than the estimated LOQ_abs_ for the 60× dilution for the 6-plex and 7-plex assays with the original protocol and with modification (2) (i.e., reduced reaction volume; Supplementary Table S19). For the 240× dilution, the observed copy numbers were lower than the estimated LOD_abs_ for the 6-plex and 7-plex assays in combination with the DG32 protocol (i.e., modification (1)), and also for the 7-plex assay with the alternative thermal cycler protocol (i.e., modification (3); Supplementary Table S19). However, no negative replicates were observed (Table 6 and Supplementary Table S19). Furthermore, all of the reactions were positive, which again indicated that the LOD_abs_ is considerably lower than 20 copies/reaction (Table 6). Taken together, the three multiplex ddPCR assays demonstrated satisfactory robustness.

### Practicabillity

The cost-efficiency of applying standard-curve-based single-event specific qPCRs for routine GMO detection has been challenged almost since the first methods were described^30^. Numerous multiplexing methods have been described, but very few of these have become established in routine analyses for various reasons. A common problem with qPCR multiplex methods is that they can be applied for qualitative analysis only, and not for quantification. Other problems include the need to handle amplified DNA for post-amplification detection and identification, the limited number of detection channels, and the potential interference between fluorescence labels in probe-based assays. The first really successful multiplex qPCR assays for GMO detection were qualitative element screening assays that were developed in 2009^30^.

To determine the cost-effectiveness of the multiplex ddPCRs, a comparison was made between: (i) applications of the multiplex ddPCR; (ii) representative screening with qPCR; and (iii) GM-event–specific qPCRs, for detection of GM soybean lines (Table 7). In these comparisons, a premise was that positive results in screening, were followed by identification and quantification of individual GM-events. The qualitative 5-plex qPCR^13^ commonly used for screening covers only six of the 11 authorised soybean lines. For the remaining five, individual identification was needed alongside the 5-plex screening. For this reason, calculations were carried out to compare multiplex ddPCR assays to this most common approach. To evaluate the cost-effectiveness of the multiplex ddPCR assays against the screening approach, comparisons were carried out for four different scenarios: (1) 5-plex screening with qPCR^13^ that yielded consistently negative results; (2) presence of only the most commonly detected GM soybean line (MON40-3-2); (3) presence of the four most common soybean lines (MON40-3-2, MON89788, A2704-12, MON87701); and (4) direct quantification of 11 authorised GM soybeans (Table 7). Comparisons were carried out for simultaneous processing of one, five or 10 samples. All of the comparisons were relative, which means that the price of the direct quantification with multiplex ddPCR was set as 100, and the other prices were calculated relative to the ddPCR.

**Table 7:** Cost-effectiveness of the developed multiplex ddPCR assays.

System	Tested samples (n)	Relative final price/sample	Hands on time (including analysis) (h)	96-well plates required (n)
Direct quantification of 11 EU authorised GM soybean lines with multiplex ddPCR assays (6-plex and 7-plex)	1	100	4	1
	5	100	7	1
	10	100	8	1
Initial screening with qPCR + identification of five transgenic soybean lines, all negative	1	100	5	2
	5	110	7	3
	10	92	11	5
Initial screening with qPCR + identification of five transgenic soybean lines PLUS quantification of one positive line from screening (5-plex)	1	153	8	3
	5	173	13	5
	10	145	19	9
Initial screening with qPCR + identification of five transgenic soybean lines PLUS quantification of three positive lines from screening (5-plex) + one positive line from identification	1	232	10	5
	5	279	18	9
	10	258	30	17
Direct quantification of 11 GM soybean lines	1	368	14	7
	5	445	31	18
	10	483	63	36

As expected, the multiplex ddPCR approach became considerably more cost-effective (from 45% to 383%) in comparison to qPCR where a sample was positive for at least one GM-events (Table 7). The costs were comparable when the sample was negative, although here the time (in hours) needed to achieve the final analytical result was longer for the qPCR analysis (Table 7). This arose because up to 10 samples can be analysed in a single 96-well plate in multiplex ddPCR, while when qPCR analysis is considered (with the exception of the initial screening and identification of five transgenic lines for one sample), it requires from two (scenario 1, one sample) up to 36 (scenario 4, 10 samples) 96-well plates (Table 7). Therefore, while up to 10 samples (one 96-well plate) can be analysed in 1 day (8 h) with the multiplex ddPCR assay, up to 8 days are needed for the qPCR assays (Table 7). Multiplexing here is thus more cost-effective and has higher throughput.

Recently, a multiplex ddPCR assay for detection and quantification of EU-authorised GM-events in maize was reported^8^. The multiplex ddPCR assays first reported in the present study complement this maize assay. The soybean multiplex ddPCR assays were approved by the Slovene accreditation body, and are included among the methods under ISO 17025 accreditation at NIB. This type of multiplexing is very useful for quantitative detection of GMOs, and potentially also for qualitative and quantitative screening for the presence of specific groups of GMOs.

## Methods

### Test materials

Certified reference materials (CRMs) were purchased from the Institute for Reference Materials and Measurements, Geel, Belgium (IRMM) and the American Oil Chemists’ Society (AOCS), and used for all 15 targeted soybean GM-events. These CRMs are consistently certified for mass/mass (m/m) GM soybean/wild-type soybean ratios. A list of all of the CRMs used in the present study is provided in Supplementary Table S20. Routine diagnostic samples that contained soybean events and samples from the USDA/GIPSA proficiency testing^31^ programme were also used in this study (Supplementary Table S13).

### DNA extraction and purification

DNA was extracted and purified from 200 mg starting material for all of the samples, using a cetyltrimethyl-ammonium bromide (CTAB) protocol, with RNase-A and proteinase-K for removal of RNA and protein from the samples, respectively (as described in Annex A.3 of ISO21570:2005^32^), with small modifications as described in Dobnik *et al*. (2015)^8^. Dilutions of the extracted stock DNA solutions were made in nuclease-free and protease-free water (Sigma-Aldrich Chemie GmbH, Munich, Germany). All of the DNA extracts and samples were stored at −20 °C until further use.

### Interactions between primers and probes

All primers and probes were investigated for potential interactions through alignment with Autodimer^28^. Autodimer makes ungapped local alignments of all primers and probes in a multiplex PCR assay, giving +1 as a reward for a match, and −1 as a penalty for a mismatch. As recommended, a score of 7 was considered as a significant interaction.

### *In-silico* multiplex specificity prediction

The specificity of the multiplex assays was assessed using electronic PCR (ePCR)^29^, the programme that runs primer-BLAST on the web^33^. The *in-silico* specificity of PCRs is usually monitored by alignment of primers to a sequence database; e.g., using Primer-BLAST^33^. In a multiplex assay, any combination of two primers can generate a PCR product. For 6-plex, 7-plex and 11-plex assays, there are 72, 98 and 242 possible combinations of primer pairs, respectively. A custom python script called ePCR multiplex was written to generate all of the combinations of these primers in the format specified for ePCR. The script is deposited at github (https://github.com/karinlag/ePCR_multiplex). The ePCRs were run for each multiplex assay with the complete insert and flanking sequences of a total of 19 GM-soybean events (Supplementary Table S2) combined with the genomes of soybean (RefSeq: GCF_000004515.3) and maize (RefSeq: GCF_000005005.1), while allowing for one mismatch for each primer, but no gaps.

### Preparation of primer and probe mixes

To facilitate the direct comparison of quantities of GM-events and the endogene, all of the probes for the GM-events were labelled with 6-carboxyfluorescein (FAM), and the probe for the lectin *Le*1 endogene was labelled with 5′-hexachloro-fluorescein phosphoramidite (HEX; Supplementary Table S21). For the estimation of the relative quantity of GM-soybean events, the *Le*1 gene was used as the endogenous reference gene. For quantification of GM soybean lines, GM-event–specific qPCR modules were used. Primer and probe sets were prepared for the simplex, duplex and multiplex reactions. A total of 15 primer and probe sets were prepared for the simplex reactions that targeted the individual soybean GM-event. A primer and probe set was also prepared for *Le*1. In total, 15 sets for duplex reactions were prepared, each of which contained the primers and probe for *Le*1 and the primers and probe of one of the 15 individual GM-events. The preparation of 6-plex, 7-plex and 11-plex primer and probe mixes was carried out as follows. For 6-plex, the primers and probes for *Le*1 and the five GM-events of MON87701, 305423, MON87708, BPS-CV127-9 and MON87769 were mixed (for final concentrations, see Supplementary Table S21). For 7-plex, the primers, and probes for *Le*1 and the six GM-events of MON40-3-2, A5574-127, A2704-12, 356043, MON89788 and MON87705 were mixed (for final concentrations, see Supplementary Table S21). For 11-plex, the same primers and probes were used as for 7-plex, with the addition of primers and probes for the four soybean GM-events of FG72, DAS-44406-6, DAS-81419-2 and DAS-68416-4. All primers and probes were purchased from Eurofins MWG Operon (Ebersberg, Germany) or from Integrated DNA Technologies (Leuven, Belgium). Primers and probes were shipped lyophilised and diluted in nuclease-free and protease-free water (Sigma-Aldrich Chemie GmbH, Munich, Germany) upon receipt.

### Droplet digital PCR and data analysis

All ddPCRs (i.e., simplex, duplex, multiplex) were performed by mixing 10 μL 2 × ddPCR Supermix for probe (no dUTP) (Bio-Rad, Pleasanton, CA, USA), 6 μL of the corresponding 6-plex, 7-plex or 11-plex primer and probe mix, and 4 μL DNA template (or 4 μL nuclease-free and protease-free water for the non-template control), in a 20 μL reaction volume. Droplets were generated in DG8 cartridges, using a QX100 Droplet Digital System droplet generator (Bio-Rad). Water-in-oil emulsions were transferred to a 96-well plate and amplified in a T100 PCR cycler (Bio-Rad). The thermal cycling conditions were: 10 min at 95 °C, followed by 40 cycles of a two-step thermal profile (15 s at 95 °C and 60 s at 60 °C, at a ramp rate of 2.5 °C/s), and final incubation at 98 °C for 10 min, followed by cooling to 4 °C. After the thermal cycling, the plates were transferred to a QX100 Droplet Digital System droplet reader (Bio-Rad). Data acquisition and analysis was performed using the QuantaSoft software, version 1.6.6 (Bio-Rad). Positive droplets (Supplementary Fig. S1) that contained the amplification products were discriminated from negative droplets (Supplementary Fig. S2) without the amplification products by applying a fluorescence amplitude threshold. The threshold was set manually (for channel 1 [FAM] at amplitudes of approximately 13200, 9300 and 18500 for 6-plex, 7-plex and 11-plex, respectively, and for channel 2 [HEX] at amplitudes of approximately 1600, 1700 and 2000 for 6-plex, 7-plex and 11-plex, respectively), using both fluorescence amplitude *versus* event number (i.e., one-dimensional amplitude), and histogram of events *versus* amplitude data streams on both the FAM and 2′-chloro-7′-phenyl-1,4-dichloro-6-carboxyfluorescein (VIC) channels (i.e., two-dimensional amplitude). The data generated by the QX100 droplet reader were rejected from subsequent analysis if a clog was detected by the Quantasoft software or if a low number of droplets (<8000) was measured per 20 μL PCR. After exporting the data, they were further analysed using Microsoft Excel spreadsheets. A digital ‘Minimum Information for Publication of Quantitative Real-Time PCR Experiments’ (MIQE) checklist is available in Supplementary Table S22, and the list for the compliance of the data with the minimum performance parameters for method acceptance is available in Supplementary Table S23.

### Comparison of simplex, duplex and multiplex reactions

The performances of the 6-plex, 7-plex and 11-plex ddPCRs were compared to both simplex and duplex assays. All of the comparisons were carried out using data obtained with duplicate analyses of DNA samples obtained from single CRMs in two dilutions (i.e., four data points). The mean copy numbers determined by simplex, duplex, 6-plex and 11-plex analyses of single CRM-derived DNA samples were used to establish the assigned concentrations (copies/µL) for the preparation of dilutions and DNA mixes containing multiple events.

### Dynamic range, repeatability, and limits of detection and quantification

A dilution series in terms of target copies was prepared by mixing DNA from all 15 targeted transgenic soybean GM-events. Each GM-event was assigned with 128 target copies/µL, except for A2704-12 and MON89788, which had 49 and 92 copies/µL, respectively. The quantity of each soybean GM-event in the DNA mix was verified by duplex ddPCR (individual GM-event and *Le*1 in the same reaction; Supplementary Table S5). The verified values for the DNA mix were used to calculate the assigned copy numbers of the targets in a dilution series of the DNA mix (referred to as DNA mixes 1–10, with DNA mixes 2–10 representing 6×, 12×, 60×, 120×, 240×, 600×, 1200×, 6000× and 12000× dilutions of DNA mix 1, respectively). The final assigned values of the mixed DNA solutions were from 2527 to 0.02 copies of GM-event targets covered by the 6-plex, from 2761 to 0.05 copies of GM-event targets covered by the 7-plex, and from 4752 to 0.04 copies of GM-event targets covered by the 11-plex. The dilution series for all three multiplexes contained from 26789 to 0.07 copies of the endogene *Le*1. Twelve replicates of the dilution series were measured by ddPCR (three separate runs, in 3 days, each containing four replicates) for the 6-plex and 11-plex, and 14 replicates of the dilution series (three separate runs, in 3 days, with the first two days containing four replicates, and the last day containing six replicates) for the 7-plex. The absolute (copy number) limit of quantification (LOQ_abs_) and detection (LOD_abs_) for ddPCR were determined based on these experimental results. Another dilution series was prepared in terms of the GM-event target content, where DNA mix 1 was diluted in non-GM soybean DNA to obtain final concentrations from 0.005% to 2% of the GM-event target relative to *Le*1 (Supplementary Table S11). Three replicates of this dilution series were measured by ddPCR for each multiplex. The relative (%) limit of quantification (LOQ_rel_) and detection (LOD_rel_) for ddPCR were determined based on these experimental results. The LOQ (both absolute and relative) was determined as the lowest concentration in a series of concentrations that consistently yielded a relative standard deviation (RSD) <25%^26, 27^. The LOD (both absolute and relative) was determined as the lowest concentration in a series of concentrations that consistently yielded positive signals with all replicates^26, 27^.

### Fitness for purpose and inter-laboratory comparison

To determine whether the new assays were fit for purpose, two different samples from the USDA/GIPSA proficiency-testing programme^31^ and samples obtained from routine diagnostics at NIB (Supplementary Table S13) were tested with all three multiplex assays. To obtain robust means of the GM-event content, the values per event reported in the proficiency-test reports were used to calculate the sum of all of the present GM-events, and finally to calculate the standard deviation. For routine diagnostic samples, the additive concentrations of the soybean GM-events were calculated based on the results of the quantification with GM-event–specific qPCRs performed at NIB, under ISO 17025 accreditation. However, the quantitative qPCR results were not always available for all of the detected GM-events in a sample. Complementary simplex ddPCRs were performed in these cases to obtain the quantitative data for all of the GM-events detected as present in a sample. All of the proficiency-test samples and routine samples were tested on two different days, and for each day at two concentrations and with two replicates (altogether eight data points per sample/assay). The inter-laboratory comparisons were carried out by transfer of protocols and samples to the NVI. Only the 6-plex and 7-plex assays were tested at the NVI, using the proficiency-test samples and routine samples (all except G158/15 and 49/15).

### Robustness

The robustness of the ddPCR multiplex assays was evaluated by comparison of the original protocol to three modified protocols. These included the use of the following: (1) automated droplet generator (AutoDG; Bio-Rad); (2) lower reaction volume (18 μL instead of 20 μL), and (3) PCR cycler with slower ramp rate (GeneAmp 9700 PCR cycler; Applied BioSystems, Foster City, CA, USA). Two samples from the repeatability experiments, one close to the LOQ (DNA mix 60× dilution) and one close the LOD (DNA mix 240× dilution) were tested in triplicate in the robustness experiments.

## Electronic supplementary material

Supplementary Information
