# Author Correction: Development and inter-laboratory assessment of droplet digital PCR assays for multiplex quantification of 15 genetically modified soybean lines

**DOI:** 10.1038/s41598-018-37135-z

**Published:** 2019-03-06

**Authors:** Alexandra Bogožalec Košir, Bjørn Spilsberg, Arne Holst-Jensen, Jana Žel, David Dobnik

**Affiliations:** 1 Department of Biotechnology and Systems Biology, National Institute of Biology, Večna pot 111, SI-1000 Ljubljana, Slovenia; 2 Josef Stefan International Postgraduate School, Jamova 39, SI-1000 Ljubljana, Slovenia; 3 Norwegian Veterinary Institute, P.O. box 750 Sentrum, 0106 Oslo, Norway

Correction to: *Scientific Reports* 10.1038/s41598-017-09377-w, published online 17 August 2017

The Supplementary Information file that accompanies this Article contains an error. In Table S21, for 7-plex MON40-3-2, the sequence “5′-FAM-CCTTTTCCATTTGGG-BHQ-3′” should read “5′-FAM-CCTTTTCCATTTGGG- MGBNFQ-3′”

