# Supplementary material for: Development and inter-laboratory assessment of droplet digital PCR assays for multiplex quantification of 15 genetically modified soybean lines
Source: Sci Rep. 2017 Aug 17;7:8601. doi: 10.1038/s41598-017-09377-w (PMC5561262; doi:10.1038/s41598-017-09377-w)
Supplement: Supplementary file 1 — Supplementary Information [file 41598_2017_9377_MOESM1_ESM.pdf]

Development and inter-laboratory assessment of droplet digital PCR assays for multiplex quantification of 15 genetically modified soybean lines

Alexandra Bogožalec Košir[1, 2, ‡, *], Bjørn Spilsberg[3, ‡], Arne Holst-Jensen[3], Jana Žel[1], David Dobnik[1]

[1]Department of Biotechnology and Systems Biology, National Institute of Biology, Večna pot 111, SI-1000 Ljubljana, Slovenia.

[2]Josef Stefan International Postgraduate School, Jamova 39, SI-1000 Ljubljana, Slovenia.

[3]Norwegian Veterinary Institute, P.O. box 750 Sentrum, 0106 Oslo, Norway.

‡ Authors contributed equally to this study.

***Correspondence should be addressed to: Alexandra Bogožalec Košir**

Department of Biotechnology and Systems Biology

National Institute of Biology

Večna pot 111

SI-1000 Ljubljana, Slovenia

Tel: +386-5-9232845

Email: alexandra.bogozalec@nib.si

Table of contents:

Table S1. Soybean GM-events authorised and in the process of authorisation for food and feed use in the EU (to September 2016). 4

Table S2. Soybean GM-events used in the ePCR....5

Table S3. Certified reference material characterisation in copies per reaction. 6

Table S4. DNA mix dilutions and their assigned copy numbers per reaction for each multiplex. 7

Table S5. Verification of assigned copy numbers per µL for each individual transgene in DNA mix 1, with duplex ddPCR. 8

Table S6. Bias between summed up assigned and experimental (duplex ddPCR verified) transgene and endogene copy numbers per reaction in DNA mix 1. 9

Table S7. Absolute limit of detection (LOD_{abs} bold), absolute limit of quantification (LOQ_{abs} underlined), and relative standard deviation (RSD) for the 6-plex assay. 10

Table S8. Absolute limit of detection (LOD_{abs} bold), absolute limit of quantification (LOQ_{abs} underlined), and relative standard deviation (RSD) for the 7-plex assay. 11

Table S9. Absolute limit of detection (LOD_{abs} bold), absolute limit of quantification (LOQ_{abs} underlined), and relative standard deviation (RSD)) for the 11-plex assay. 12

Table S10. Absolute limit of detection (LOD_{abs} bold), absolute limit of quantification (LOQ_{abs} underlined), and relative standard deviation (RSD) for *Le*1 endogene.... 13

Table S11. DNA mixes I to VII, assigned overall copy number and copy number for each multiplex... 14

Table S12. Relative limit of detection (LOD_{rel} bold) and quantification (LOQ_{rel} underlined), for all three multiplex assays. 15

Table S13. Bias between assigned and experimental endogene copy numbers per reaction.... 16

Table S14. Routine and USDA proficiency programme samples.... 17

Table S15. Inter-laboratory comparison of combined results for 6-plex and 7-plex ddPCR assay for genetically modified soybean quantification. 18

Table S16. Inter-laboratory comparison of results for 6-plex ddPCR assay for genetically modified soybean quantification.... 19

Table S17. Inter-laboratory comparison of results for 7-plex ddPCR assay for genetically modified soybean quantification.... 20

Table S18. Accuracy (trueness and precision) of the ddPCR assays.... 21

Table S19. Robustness test: comparison of copy numbers.... 23

Table S21. Primers and probes used in this study.... 25

Table S22. MIQE checklist. 27

Table S23. Compliance with minimum performance parameters for methods acceptance according to ref. 26[a] 32

Figure S1. Example of positive experimental results. Fluorescence of droplets in a positive sample (6-plex assay). 38
Figure S2. Example of negative experimental results. 39

Table S1. Soybean GM-events authorised and in the process of authorisation for food and feed use in the EU (to September 2016).

Line/species	Unique identifier	Legal status
A2704-12	ACS-GMØØ5-3	Authorised
MON89788	MON-89788-1	Authorised
MON40-3-2	MON-Ø4Ø32-6	Authorised
356043	DP-356Ø43-5	Authorised
A5547-127	ACS-GMØØ6-4	Authorised
MON87705	MON-877Ø5-6	Authorised
MON87701	MON-877Ø1-2	Authorised
MON87708	MON-877Ø8-9	Authorised
MON87769	MON-87769-7	Authorised
305423	DP-3Ø5423-1	Authorised
BPS-CV127-9	BPS-CV127-9	Authorised
MON87701 x MON89788	MON-877Ø1-2 x MON-89788-1	Authorised
FG72*	MST-FGØ72-2	Authorised
MON 87705 x MON 89788	MON-877Ø5-6 × MON-89788-1	Authorised
MON 87708 x MON 89788	MON-877Ø8-9 x MON-89788-1	Authorised
DAS-68416-4	DAS-68416-4	Valid EFSA application
FG72*	MST-FGØ72-2	Valid EFSA application
DAS-44406-6	DAS-444Ø6-6	Valid EFSA application
DAS-81419-2	DAS-81419-2	Valid EFSA application
305423 x 40-3-2	DP-305423-1 x MON-04032-6	Valid EFSA application
MON 87769 x MON 89788	MON-87769-7 × MON-89788-1	Valid EFSA application
MON 87705 x MON 89788	MON-877Ø5-6 × MON-89788-1	Valid EFSA application
MON 87708 x MON 89788	MON-877Ø8-9 x MON-89788-1	Valid EFSA application
MON 87705 x MON 87708 x MON89788*	MON-877Ø5-6 × MON-877Ø8-9 x MON-89788-1	Valid EFSA application

* On July 22, after the presented experimental work was completed, FG72 was authorised, together with two hybrid stacks, MON 87705 × MON 89788 and MON 87708 × MON 89788, additionally for the stack MON 87705 x MON 87708 x MON89788 a valid EFSA application was submitted.

Table S2. Soybean GM-events used in the ePCR.

Soybean GM-events	GenBank accession
A2704-12*	CS447634
A2704-12**	CS447635
A5547-127	CS444252
BPS-CV127-9	HD112081
DAS-44406-6	JC006926
DAS-68416-4	JB090933
DAS-81419-2	JC006928
305423	GM037889
356043	FB727428
Event1606	HK037693
FG72	HK747743
MON40-3-2	HW479040
MON87712	HW503815
MON87701	HB386793
MON87705	JA486740
MON87708	JB048350
MON87754	HK762611
MON87769	HB976128
MON89788	FB572992
SYHT0H2	JC050419

*3’ junction between the host and insert, not including the target for QT-EVE-GM-004

**5’ junction between the host and insert, not including the target for QT-EVE-GM-004

Table S3. Certified reference material characterisation in copies per reaction.

**Soybean GM-events**	**Mean copy number**			
	**Simplex**	**Duplex**	**Multiplex**	**Mean**
MON87701	329548	312255	246741	296181
*Le*1	333848	323910	261405	306387
MON87708	170792	186888	170838	176172
*Le*1	165436	183505	167695	172212
MON87769	2574882	2409375	2441373	2475210
*Le*1	2613941	2544248	2562539	2573576
305423	23491	31063	25673	26742
*Le*1	298139	358422	314061	323541
BPS-CV127-9	235109	233961	213966	227679
*Le*1	397970	425314	404040	409108
A2704-12	422058	390567	361810	391478
*Le*1	411862	414867	381314	402681
MON89788	130258	122812	103137	118736
*Le*1	138150	131760	114036	127982
MON40-3-2	46314	36459	27697	36823
*Le*1	426855	392449	279546	366283
356043	54104	48933	35155	46064
*Le*1	534809	444045	331342	436732
MON87705	320407	345418	302935	322920
*Le*1	336037	346189	309110	330445
A5547-127	316956	335020	289262	313746
*Le*1	312398	354759	311134	326097
DAS-68416-4	46390	45262	28432	40028
*Le*1	410511	381669	272252	354811
FG72	345804	331541	315753	331032
*Le*1	325130	322551	334878	327519
DAS-44406-6	230476	261977	156168	216207
*Le*1	227793	226744	145001	199846
DAS-81419-2	359756	348622	287884	332087
*Le*1	368132	285900	338532	330855

Table S4. DNA mix dilutions and their assigned copy numbers per reaction for each multiplex.

DNA mix		Assigned copy number		
		**6-plex**	**7-plex**	**11-plex**
1	GM-events	2527	2761	4752
	*Le*1	26294	26294	26294
2	GM-events	421	460	792
	*Le*1	4382	4382	4382
3	GM-events	211	230	396
	*Le*1	2191	2191	2191
4	GM-events	42	46	79
	*Le*1	438	438	438
5	GM-events	21	23	40
	*Le*1	219	219	219
6	GM-events	11	12	20
	*Le*1	110	110	110
7	GM-events	4	5	8
	*Le*1	44	44	44
8	GM-events	2	2	4
	*Le*1	22	22	22
9	GM-events	0	0	1
	*Le*1	4	4	4
10	GM-events	0	0	0
	*Le*1	2	2	2

Table S5. Verification of assigned copy numbers per µL for each individual transgene in DNA mix 1, with duplex ddPCR.

Soybean GM-events	Duplex		Assigned		Bias	
	Soybean GM- event copy number	*Le*1 copy number	Soybean GM- event copy number	*Le*1 copy number	Soybean GM- event (%)	*Le*1
MON87701	128	6914	128	6570	0.44	5.23
MON87708	124	6875	128	6570	-3.15	4.63
MON87769	161	6888	128	6570	26.24	4.84
305423	112	6677	128	6570	-12.19	1.62
BPS-CV127-9	106	6449	128	6570	-17.28	-1.85
A2704-12	43	6342	49	6570	-13.84	-3.48
MON89788	81	6410	92	6570	-12.14	-2.45
MON40-3-2	156	6626	128	6570	21.63	0.85
356043	150	6868	128	6570	17.37	4.53
A5547-127	126	7406	128	6570	-1.72	12.71
MON87705	135	6781	128	6570	5.86	3.20
DAS68416	109	6590	128	6570	-14.88	0.30
FG72	137	6415	128	6570	6.82	-2.37
DAS44406	135	6731	128	6570	5.73	2.44
DAS81419	117	6487	128	6570	-8.44	-1.27

Table S6. Bias between summed up assigned and experimental (duplex ddPCR verified) transgene and endogene copy numbers per reaction in DNA mix 1.

	**6-plex**			**7-plex**			**11-plex**		
	**Experimental copy number**	**Assigned copy number**	**Bias (%)**	**Experimental copy number**	**Assigned copy number**	**Bias (%)**	**Experimental copy number**	**Assigned copy number**	**Bias (%)**
GM-event	2527	2557	-1.19	2761	2612	5.69	4752	4658	2.01
*Le*1	27042	26282	2.89	26955	26282	2.56	26662	26282	1.45
GM-events (%)	9.34	9.73	-3.97	10.24	9.94	3.05	17.82	17.72	0.55

Table S7. Absolute limit of detection (LOD_{abs} **bold**), absolute limit of quantification (LOQ_{abs} underlined), and relative standard deviation (RSD) for the 6-plex assay.

DNA mix	Assigned copy number	Repeat 1		Repeat 2		Repeat 3		All		Bias to assigned copy number (%)
		Mean copy number	RSD (%)	Mean copy number	RSD (%)	Mean copy number	RSD (%)	Mean copy number	RSD (%)	
1	2557	2176	5.59	2252	4.49	2260	6.16	2229	5.41	-12.81
2	426	312	13.82	353	9.53	328	8.64	331	10.66	-22.34
3	213	185	7.86	194	3.23	189	9.20	189	6.76	-11.15
4	43	32	11.36	44	18.61	39	7.99	38	12.65	-10.23
5	21	18	32.91	20	31.26	16	30.59	18	31.58	-13.38
6	11	**6**	**23.00**	**10**	**28.40**	7	28.37	**8**	**26.59**	**-26.60**
7	4	5 [a]	ND [b]	6 [a]	ND [b]	**5**	**16.73**	5 [a]	ND [b]	ND [b]
8	2.1	Neg.	Neg.	Neg.	Neg.	6 [a]	ND [b]	5 [a]	ND [b]	ND [b]
9	0.4	Neg.	Neg.	Neg.	Neg.	Neg.	Neg.	Neg.	Neg.	Neg.
10	0.2	Neg.	Neg.	Neg.	Neg.	Neg.	Neg.	Neg.	Neg.	Neg.

[a] At least one replicate was negative.

[b] ND, not determined because of negative replicate(s).

Neg., all replicates were negative

Table S8. Absolute limit of detection (LOD_{abs} **bold**), absolute limit of quantification (LOQ_{abs} underlined), and relative standard deviation (RSD) for the 7-plex assay.

**DNA mix**	**Assigned copy number**	**Repeat 1**		**Repeat 2**		**Repeat 3**		**All**		**Bias to assigned copy number (%)**
		**Mean copy number**	**RSD (%)**	**Mean copy number**	**RSD (%)**	**Mean copy number**	**RSD (%)**	**Mean copy number**	**RSD (%)**	
1	2612	2525	1.79	2441	5.80	2418	9.10	2429	6.46	-6.99
2	435	384	2.19	392	15.21	390	4.97	391	9.39	-10.22
3	218	185	10.87	194	10.36	178	7.78	186	9.72	-14.56
4	44	33	5.99	42	18.62	41	9.16	39	15.28	-10.64
5	22	27	27.02	23	28.20	22	20.91	22	25.90	1.59
6	11	**10**	**11.96**	**11**	**27.79**	**9**	**40.48**	**10**	**30.20**	**-8.48**
7	4	5[a]	ND[b]	3[a]	ND[b]	3[a]	ND[b]	3[a]	ND[b]	ND[b]
8	2.2	Neg.	Neg.	Neg.	Neg.	Neg.	Neg.	Neg.	Neg.	Neg.
9	0.4	Neg.	Neg.	Neg.	Neg.	Neg.	Neg.	Neg.	Neg.	Neg.
10	0.2	Neg.	Neg.	Neg.	Neg.	Neg.	Neg.	Neg.	Neg.	Neg.

[a] At least one replicate was negative.

[b] ND, not determined because of negative replicate(s).

Neg., all replicates were negative

Table S9. Absolute limit of detection (LOD_{abs} **bold**), absolute limit of quantification (LOQ_{abs} underlined), and relative standard deviation (RSD)) for the 11-plex assay.

DNA mix	Assigned copy number	Repeat 1		Repeat 2		Repeat 3		All		Bias to assigned copy number (%)
		Mean copy number	RSD (%)	Mean copy number	RSD (%)	Mean copy number	RSD (%)	Mean copy number	RSD (%)	
1	4658	3943	3.06	3535	6.74	3422	12.00	3633	7.27	-22.00
2	776	583	8.66	574	5.08	564	7.49	574	7.08	-26.11
3	388	352	9.27	278	12.70	319	8.40	316	10.12	-18.54
4	78	66	11.62	78	6.15	61	3.50	68	7.09	-11.98
5	39	41	7.33	41	11.54	35	16.37	39	11.76	0.09
6	19	19	51.41	22	12.81	**14**	**45.85**	**18**	**36.69**	**-5.89**
7	8	9	27.89	**15**	**40.47**	9 [a]	ND[b]	12	34.18	59.57
8	3.9	**6**	**26.86**	8 [a]	ND[b]	Neg.	Neg.	7 [a]	ND[b]	ND[b]
9	0.8	9 [a]	ND[b]	Neg.	Neg.	Neg.	Neg.	Neg.	Neg.	Neg.
10	0.4	Neg.	Neg.	Neg.	Neg.	Neg.	Neg.	Neg.	Neg.	Neg.

[a] At least one replicate was negative.

[b] ND, not determined because of negative replicate(s).

Neg., all replicates were negative

Table S10. Absolute limit of detection (LOD_{abs} **bold**), absolute limit of quantification (LOQ_{abs} underlined), and relative standard deviation (RSD) for *Le*1 endogene.

DNA mix	Assigned copy number	Repeat 1		Repeat 2		Repeat 3		All		Bias to assigned copy number (%)
		Mean copy number	RSD (%)	Mean copy number	RSD (%)	Mean copy number	RSD (%)	Mean copy number	RSD (%)	
1	26788	23313	4.17	23207	6.26	22189	7.26	22903	2.71	-12.79
2	4465	3282	6.97	3510	6.26	3355	4.70	3382	3.44	-22.73
3	2232	1859	7.51	1874	6.24	1760	4.48	1831	3.37	-16.33
4	446	380	8.98	401	9.25	352	4.68	378	6.42	-13.69
5	223	202	13.16	201	9.84	180	9.90	194	6.33	-11.23
6	112	92	14.21	85	13.70	85	11.33	87	4.58	-20.25
7	45	40	21.56	34	19.00	35	12.33	36	8.65	-17.17
8	22.3	**16**	**29.68**	**21**	**23.59**	**14**	**32.32**	**17**	20.19	**-21.87**
9	4.5	6 [a]	ND[b]	3 [a]	ND[b]	5 [a]	ND[b]	5 [a]	ND[b]	ND[b]
10	2.23	Neg.	Neg.	Neg.	Neg.	Neg.	Neg.	Neg.	Neg.	Neg.

[a] At least one replicate was negative.

[b] ND, not determined because of negative replicate(s).

Neg., all replicates were negative

Table S11. DNA mixes I to VII, assigned overall copy number and copy number for each multiplex.

DNA mix	Assigned overall[a]		Assigned 6-plex		Assigned 7-plex		Assigned 11-plex	
	GM-event (%)	Copy number	GM-event (%)	Copy number	GM-event (%)	Copy number	GM-event (%)	Copy number
I	2	1000	0.80	333	1.33	400	1.33	667
II	0.9	450	0.36	150	0.60	180	0.60	300
III	0.5	250	0.20	83	0.33	100	0.33	167
IV	0.1	50	0.04	17	0.07	20	0.07	33
V	0.05	25	0.02	8	0.03	10	0.03	17
VI	0.02	10	0.008	3	0.013	4	0.013	7
VII	0.005	2.5	0.002	1	0.003	1	0.003	2

[a] assigned number of copies for *Le*1 is 50000, for all dilutions

Table S12. Relative limit of detection (LOD_{rel} **bold**) and quantification (LOQ_{rel} underlined), for all three multiplex assays.

Assay	DNA mix	Assigned[a]		Experimental				Bias (%)	
		GM-event (%)	Copy number	Mean GM-event (%)	RSD (%) GM-event	Mean copy number	RSD (%) copy number	GM-event (%)	Copy number
**6-plex**	I	0.67	333	0.73	6.12	355	5.49	9.50	6.50
	II	0.30	150	0.36	21.07	172	5.85	19.67	14.67
	III	<u>0.17</u>	<u>83</u>	<u>0.20</u>	<u>3.14</u>	<u>96</u>	<u>13.08</u>	<u>18.20</u>	<u>15.20</u>
	IV	0.03	17	0.04	46.79	20	44.80	26.00	20.00
	V	**0.017**	**8**	**0.015**	**59.66**	**7**	**64.21**	**-10.00**	**-16.00**
	VI	0.007	3	0.001	173.21	1[b]	ND[c]	ND[c]	ND[c]
	VII	0.002	1	0.002	173.21	1[b]	ND[c]	ND[c]	ND[c]
**7-plex**	I	0.80	400	0.82	5.72	418	8.57	2.00	4.50
	II	0.36	180	0.42	5.47	212	6.42	17.50	17.78
	III	<u>0.20</u>	<u>100</u>	<u>0.21</u>	<u>11.36</u>	<u>107</u>	<u>12.94</u>	<u>3.50</u>	<u>7.00</u>
	IV	0.04	20	0.05	35.49	24	34.72	22.50	20.00
	V	**0.02**	**10**	**0.017**	**18.92**	**9**	**13.26**	**-15.00**	**-10.00**
	VI	0.008	4	0.007	108.75	3[b]	ND[c]	ND[c]	ND[c]
	VII	0.002	1	0.008	144.35	4[b]	ND[c]	ND[c]	ND[c]
**11-plex**	I	1.33	667	1.49	14.22	704	11.71	11.49	5.62
	II	0.60	300	0.68	12.97	333	16.83	13.02	11.12
	III	<u>0.33</u>	<u>167</u>	<u>0.44</u>	<u>19.97</u>	<u>210</u>	<u>20.49</u>	<u>31.64</u>	<u>25.95</u>
	IV	0.07	33	0.05	73.58	24	73.97	-20.53	-27.02
	V	0.03	17	0.02	38.62	10	41.11	-40.44	-42.60
	VI	**0.013**	**7**	**0.028**	**48.91**	**12**	**49.41**	**107.49**	**77.54**
	VII	0.003	2	0.015	102.44	7[b]	ND[c]	ND[c]	ND[c]

[a] assigned number of copies for *Le*1 is 50000, for all dilutions.

[b] At least one replicate was negative.

[c] ND, not determined due to negative replicate(s).

Table S13. Bias between assigned and experimental endogene copy numbers per reaction.

DNA mix	Assigned copy number	6-plex		7-plex		11-plex		Mean copy number	Bias (%)
		Experimental copy number	Bias (%)	Experimental copy number	Bias (%)	Experimental copy number	Bias (%)		
I	50000	48696	-2.61	51283	2.57	47392	-5.22	49124	-1.75
II	50000	47995	-4.01	50205	0.41	49159	-1.68	49120	-1.76
III	50000	48743	-2.51	51760	3.52	47936	-4.13	49480	-1.04
IV	50000	47685	-4.63	49743	-0.51	45457	-9.09	47628	-4.74
V	50000	48083	-3.83	53565	7.13	49146	-1.71	50265	0.53
VI	50000	47012	-5.98	49793	-0.41	44921	-10.16	47242	-5.52
VII	50000	48445	-3.11	51577	3.15	46036	-7.93	48686	-2.63

Table S14. Routine and USDA proficiency programme samples.

Sample	Code	Source/ use	Total determined (%)[a]	Targeted by 6-plex (%)	Targeted by 7-plex (%)
**Real-life**	G023/11	Flour	0.91	0.00	0.91
	G071/15	Soybean meal	94.20	2.82	91.34
	G158/15	Strawberry milkshake	<0.10	0.00	<0.10
	1/15	Chicken feed	0.94	0.00	0.94
	49/15	Soybean flour	0.48[b]	0.00	0.00
	75/15	Feed mix	91.40	15.87	75.52
	116/15	Feed mix	1.68	<0.10	1.68
	226/14	PU-starter premium	2.70	0.00	2.70
**USDA**	272/14	Flour	1.29	0.07	1.22
**proficiency**	274/14	Flour	2.26	0.74	1.52

[a] Determined by qPCR under ISO 17025 accreditation

[b] 0.48 (LLP), only DAS68416, so will only be detected by 11-plex

Table S15. Inter-laboratory comparison of combined results for 6-plex and 7-plex ddPCR assay for genetically modified soybean quantification.

Sample	Assigned GM-events (%) [a]	Sum GM-events (6-plex + 7-plex) (%)		Bias (%)	
		NIB	NVI	NIB	NVI
G023/11	0.91	1.03	1.06	14.13	17.52
G071/15	94.20	91.39	91.92	-2.98	-2.42
226/14	2.70	2.42	2.47	-10.37	-8.52
272/14	1.29	1.40	1.39	8.60	7.45
274/14	2.26	2.34	2.63	3.58	16.53
1/15	0.86	0.95	1.12	2.67	20.62
75/15	91.38	90.59	96.29	-0.86	5.37
116/15	1.68	1.73	1.91	2.70	13.53

[a] Assigned GM-event (%), value determined by NIB using event-specific qPCR or simplex ddPCR methods

Table S16. Inter-laboratory comparison of results for 6-plex ddPCR assay for genetically modified soybean quantification.

Sample	GM-event (%) [a]				RSD_r% for GM-event		Mean GM-event copy number		RSD_r (%)GM-event copy number		Mean *Le*1 copy number		RSD_r (%) *Le*1 copy number	
	**Mean**	**RSD_R (%)**	**NIB**	**NVI**	**NIB**	**NVI**	**NIB**	**NVI**	**NIB**	**NVI**	**NIB**	**NVI**	**NIB**	**NVI**
G023/11	NS[b]	NS[b]	NS[b]	NS[b]	NS[b]	NS[b]	NS[b]	NS[b]	NS[b]	NS[b]	41716	46392	7.23	5.96
G071/15	2.64	3.08	2.60	2.68	13.50	22.02	1633	1654	14.13	24.31	62870	60887	4.60	5.98
226/14	ND[c]	ND[c]	NS[b]	0.05 [d]	NS[b]	185.67 [d]	NS[b]	8 [e, d]	NS[b]	185.50 [d]	15927	15929	5.32	2.80
272/14	0.06		0.07	0.05	1.51	17.28	82	72	13.01	20.72	131216	141689	4.63	12.22
274/14	1.00	9.28	0.95	1.04	22.35	23.55	73	73	24.70	46.66	7693	8459	5.74	23.58
1/15	NS[b]	NS[b]	NS[b]	NS[b]	NS[b]	NS[b]	NS[b]	NS[b]	NS[b]	NS[b]	151429	135436	1.94	4.78
75/15	16.40	6.50	15.88	16.91	5.38	4.82	4373	4857	13.66	6.62	28930	28714	4.31	3.32
116/15	0.05	30.42	0.05	0.06	15.32	35.77	29 [e]	37 [e]	52.48 [f]	29.38	54445	54047	5.22	12.66

[a] Means observed at NIB and NVI (RSD_R, reproducibility relative standard deviation between NIB and NVI), (value observed at NIB/value observed at NVI)

[b] NS, negative sample

[c] ND, cannot be determined

[d] Six out of eight replicates negative

[e] Copy number lower than estimated limit of quantification

[f] Value not compliant with acceptance value for dynamic range (quantifiability)

Table S17. Inter-laboratory comparison of results for 7-plex ddPCR assay for genetically modified soybean quantification.

Sample	GM-event (%) [a]				RSD_r (%) for GM-event		Mean GM-event copy number		RSD_r (%) GM-event copy number		Mean *Le*1 copy number		RSD_r (%) *Le*1 cpopy number	
	Mean	RSD_R	NIB	NVI	NIB	NVI	NIB	NVI	NIB	NVI	NIB	NVI	NIB	NVI
G023/11	1.04	2.97	1.03	1.06	8.45	10.34	465	455	9.29	8.84	45050	35124	5.45	43.12 [b]
G071/15	89.02	0.51	88.79	89.24	5.22	2.38	58143	52292	4.96	5.61	65510	58593	2.57	33.66
226/14	2.39	-2.24	2.42	2.36	20.20	19.51	405	376	20.67	13.77	16825	15857	4.90	3.65
272/14	1.34	-0.25	1.34	1.33	7.57	7.86	1742	1700	4.86	13.63	131322	124938	10.83	14.37
274/14	1.49	14.72	1.39	1.59	13.95	32.84	104	135	15.33	41.78 [b]	7515	7126	11.62	49.28 [b]
1/15	1.04	17.49	0.95	1.12	5.33	6.71	1373	1410	10.36	10.59	144551	125880	12.39	10.73
75/15	77.05	6.25	74.71	79.38	17.68	4.28	22766	21228	6.84	11.92	30464	26923	6.10	12.17
116/15	1.76	9.97	1.68	1.84	9.13	14.56	916	935	10.58	20.27	54661	51585	5.78	14.55

[a] Means observed at NIB and NVI, (RSD_R = reproducibility relative standard deviation between NIB and NVI), (value observed at NIB/value observed at NVI)

[b] Value not compliant with acceptance value for dynamic range (quantifiability)

Table S18. Accuracy (trueness and precision) of the ddPCR assays.

Sample	Combined 6-plex and 11-plex			6-plex						11-plex					
	Assigned GM-event (%) [a]	Sum GM-event (%)	Bias (%)	GM-event (%)	RSD_r (%) for GM-line	Mean GM-event copy number	RSD_r (%) GM-event copy number	Mean *Le*1 copy number	RSD_r (%) for *Le*1 copy number	GM-event (%)	RSD_r (%) for GM-line	Mean GM-event copy number	RSD_r (%) GM-event copy number	Mean *Le*1 copy number	RSD_r (%) for *Le*1 copy number
G023/11	0.91	0.97	6.6	NS[b]	NS[b]	NS[b]	NS[b]	41716	7.2	0.97	6.2	410	8.66	42335	6.5
G071/15	94.2	90.23	-4.2	2.6	13.5	1633	14.13	62870	4.6	87.63	4.62	54517	3.83	62317	5.7
G158/15	<0.1	0.07	NA[c]	0.0	0.0	0.0	0.0	583852	28.5	0.07	18.94	286	15.47	448603	27.8
226/14	2.7	2.49	-7.8	0.0	0.0	0.0	0.0	15927	5.3	2.49	22.69	397	22.33	15994	4.4
272/14	1.29	1.31	1.6	0.07	1.51	82	13.01	131216	4.6	1.24	8.42	70	16.41	5706	16.2
274/14	2.26	2.89	27.9	0.95	22.35	73	24.7	7693	5.7	1.94	0.48	160	23.74	8226	6.6
1/15	0.94	1.03	9.6	0.0	0.0	0.0	0.0	151429	1.9	1.03	12.62	1472	12.92	143445	5.4
49/15	0.48	0.53	10.4	0.0	0.0	0.0	0.0	66306	13.2	0.53	0.21	342	35.31[d]	63939	14.0
75/15	91.38	91.88	0.5	15.88	5.38	4373	13.66	28930	4.3	76	4.43	17137	5.3	22548	2.4
116/15	1.68	1.92	14.3	0.05	15.32	29 [e]	52.48 [d]	54445	5.2	1.87	15.32	937	15.18	50202	3.5

[a] Assigned GM-event (%),determined by NIB using event-specific qPCR methods under ISO 17025 accreditation.

[b] NS, negative sample

[c] NA, not applicable

[d] Value not compliant with acceptance value for dynamic range (quantifiability)

[e] Copy number lower than estimated limit of quantification

Table S19. Robustness test: comparison of copy numbers.

		6-plex		7-plex		11-plex	
**Protocol**	**Dilution** / **Factor**	**60×** [a]	**240×** [b]	**60×**	**240×**	**60×**	**240×**
Original	Mean GM-event copy number/reaction	36[c]	10	34[c]	9[d]	67	12
	RSD (%) GM-event copy number/reaction	13.28	57.86[e]	9.44	31.06	13.53	24.73
	Mean *Le*1 copy number/reaction	352	71	346	75	329	76
	RSD (%) *Le*1 copy number/reaction	16.85	16.73	3.77	8.00	8.36	6.13
DG32	Mean GM-event copy number/reaction	39	6[d]	39	9[d]	80	18
	RSD (%) GM-event copy number/reaction	17.72	24.73	4.95	58.05[e]	42.39[e]	20.00
	Bias (%)to original GM-event copy number	7.69	-66.67	12.82	0.00	16.25	33.33 [f]
	Mean *Le*1 copy number/reaction	351	76	391	83	350	93
	RSD (%) *Le*1 copy number/reaction	4.66	18.45	10.77	8.46	4.87	22.58
	Bias (%) to original *Le*1 copy number	-0.28	6.58	11.51	9.64	6.00	18.28
18 µL sample	Mean GM-event copy number/reaction	30[c]	10	34[c]	11	70	16
	RSD (%) GM-event copy number/reaction	8.64	50.50[e]	8.85	13.77	11.13	32.48
	Bias (%)to original GM-event copy number	-20.00	0.00	0.00	18.18	4.29	25.00
	Mean *Le*1 copy number/reaction	367	71	373	68	388	86
	RSD (%) *Le*1 copy number/reaction	10.78	13.34	4.09	20.78	8.22	14.49
	Bias (%) to original *Le*1 copy number	4.09	0.00	7.24	-10.29	15.21	11.63
Thermal cycler	Mean GM-event copy number/reaction	42	10	40	8[d]	64	18
	RSD (%) GM-event copy number/reaction	10.49	15.94	17.30	22.51	21.16	39.86[e]
	Bias (%)to original GM-event copy number	14.29	0.00	15.00	-12.50	-4.69	33.33[f]
	Mean *Le*1 copy number/reaction	391	81	372	84	362	78
	RSD (%) *Le*1 copy number/reaction	4.45	13.46	4.84	17.13	10.84	7.99
	Bias (%) to original *Le*1 copy number	9.97	12.35	6.99	10.71	9.12	2.56

[a] 60× dilution was chosen to challenge the LOQ.

[b] 240× dilution was chosen to challenge the LOD.

[c] Copy number lower than estimated limit of quantification.

[d] Copy number lower than estimated limit of detection.

[e] RSD higher than acceptance value (35%) for dynamic range (quantifiability).

[f] Bias higher than acceptance value (25%) for dynamic range (quantifiability).

Table S20. **Certified reference material used in this study.**

Event	Available at	Reference material ID	Certified GM content (mass/mass; %)
MON87705	AOCS[a]	AOCS 0210-A	> 99.4
MON89788	AOCS	AOCS 0906-B	100
MON40-3-2	JRC-IRMM[b]	ERM-BF410gk	10
356043	JRC-IRMM	ERM-BF425d	10
A5547-127	AOCS	AOCS 0707-C2	> 99.99
A2704-12	AOCS	AOCS 0707-B4	> 99.99
MON87708	AOCS	AOCS 0311-A	> 99.05
305423	JRC-IRMM	ERM-BF526d	10
BPS-CV127-9	AOCS	AOCS 0911-C	> 96.32
MON87769	AOCS	AOCS 0809-B	> 99.94
MON87701	AOCS	AOCS 0512-A	> 99.94
DAS-68416-4	JRC-IRMM	ERM-BF432d	10
FG72	AOCS	AOCS 0610-A2	> 99.99
DAS-44406-6	JRC-IRMM	ERM-BF436b	98.60
DAS-81419-2	JRC-IRMM	ERM-BF437b	98.60

[a]AOCS (American Oil Chemist Society) and [b] JRC-IRMM (Joint Research Centre, Institute for Reference Materials and Measurements) are providers of certified reference material.

Table S21. Primers and probes used in this study.

Multiplex	Event	Forward / reverse / probe (F/ R/P)	Sequence	Amplicon length (bp)	Final concentration in PCR (nM)
Endogene	*Le*1	F	5'-CCAGCTTCGCCGCTTCCTTC-3'	74	650
	*Le*1	R	5'-GAAGGCAAGCCCATCTGCAAGCC-3'		650
	*Le*1	P	5'-HEX-CTTCACCTTCTATGCCCCTGACAC-BHQ-3'		180
6-plex	305423	F	5'-CGTGTTCTCTTTTTGGCTAGC-3'	93	800
	305423	R	5'-GTGACCAATGAATACATAACACAAACTA-3'		500
	30542	P	5'-FAM-TGACACAAATGATTTTCATACAAAAGTCGAGA-BHQ-3'		220
	MON87708	F	5'-TCATACTCATTGCTGATCCATGTAG-3'	91	300
	MON87708	R	5'-AGAACAAATTAACGAAAAGACAGAACG-3'		300
	MON87708	P	5'-FAM-TCCCGGACTTTAGCTCAAAATGCATGTA-BHQ-3'		150
	BPS-CV127-9	F	5'-AACAGAAGTTTCCGTTGAGCTTTAAGAC-3'	88	400
	BPS-CV127-9	R	5'-CATTCGTAGCTCGGATCGTGTAC-3'		400
	BPS-CV127-9	P	5'-FAM-TTTGGGGAAGCTGTCCCATGCCC-BHQ-3'		100
	MON87769	F	5'-CATACTCATTGCTGATCCATGTAGATT-3'	87	600
	MON87769	R	5'-GCAAGTTGCTCGTGAAGTTTTG-3'		600
	MON87769	P	5'-FAM-CCCGGACATGAAGCCATTTACAATTGAC-BHQ-3’		600
	MON87701	F	5'-TGGTGATATGAAGATACATGCTTAGCAT-3'	89	600
	MON87701	R	5'-CGTTTCCCGCCTTCAGTTTAAA-3'		600
	MON87701	P	5'-FAM-TCAGTGTTTGACACACACACTAAGCGTGCC-BHQ-3’		250
7-plex	MON40-3-2	F	5'-TTCATTCAAAATAAGATCATACATACAGGTT-3'	84	600
	MON40-3-2	R	5'-GGCATTTGTAGGAGCCACCTT-3'		600
	MON40-3-2	P	5'-FAM-CCTTTTCCATTTGGG-BHQ-3'		200
	A2704-12	F	5'-GCAAAAAAGCGGTTAGCTCCT-3'	64	200
	A2704-12	R	5'-ATTCAGGCTGCGCAACTGTT-3'		200
	A2704-12	P	5'-FAM-CGGTCCTCCGATCGCCCTTCC-BHQ-3'		200
	A5547-127	F	5'-GCTATTTGGTGGCATTTTTCCA-3'	75	200
	A5547-127	R	5'-CACTGCGGCCAACTTACTTCT-3'		200
	A5547-127	P	5'-FAM-CCGCAATGTCATACCGTCATCGTTGT-BHQ-3'		200
	MON89788	F	5'-TCCCGCTCTAGCGCTTCAAT-3'	139	600
	MON89788	R	5'-TCGAGCAGGACCTGCAGAA-3'		600
	MON8978	P	5'-FAM-CTGAAGGCGGGAAACGACAATCTG-BHQ-3'		200

	356043	F	5'-GTCGAATAGGCTAGGTTTACGAAAAA-3'	99	650
	356043	R	5'-TTTGATATTCTTGGAGTAGACGAGAGTGT-3'		650
	35604	P	5'-FAM-CTCTAGAGATCCGTCAACATGGTGGAGCAC-BHQ-3'		180
	MON87705	F	5'-TTCCCGGACATGAAGCCATTTAC-3'	86	450
	MON87705	R	5'-ACAACGGTGCCTTGGCCCAAAG-3'		450
	MON87705	P	5'-FAM-AAGAGACTCAGGGTGTTGTTATCACTGCGG-BHQ-3'		250
11-plex	DAS68416-4	F	5'GTACATTAAAAACGTCCGCAATGTGT-3'	130	550
(addition	DAS68416-4	R	5'GTTTAAGAATTAGTTCTTACAGTTTATTGTTAG-3'		550
to 7-plex)	DAS68416-4	P	5'-FAM-TTAAGTTGTCTAAGCGTCAATA–MGBNFQ-3'		150
	FG72	F	5'-AGATTTGATCGGGCTGCAGG-3'	70	400
	FG72	R	5'-GCACGTATTGATGACCGCATTA-3'		400
	FG72	P	5'-FAM-AATGTGGTTCATCCGTCTT-MGBNFQ-3'		200
	DAS44406-6	F	5'-TTATTGTTCTTGTTGTTTCCTCTTTAGG–3'	99	300
	DAS44406-6	R	5'-CCTCAATTGCGAGCTTTCTAATTT-3'		300
	DAS44406-6	P	5'-FAM-ATTCGGACCTCCATGATGACCTTACCGTT-BHQ-3'		180
	DAS81419-2	F	5'-TCTAGCTATATTTAGCACTTGATATTCAT-3'	105	400
	DAS81419-2	R	5'-GCTTCAAGATCCCAACTTGCG-3'		400
	DAS81419-2	P	5'-FAM-ATCAACAGGCACCGATGCGCACCG-BHQ-3'		120

Table S22. MIQE checklist.

Item	Importance	Included	Comments
**Experimental design**			
Definition of experimental and control groups	E[a]	Yes	
Number within each group	E	Yes	
Assay carried out by core laboratory or investigator's laboratory?	D[b]	Yes	Investigator's laboratory
Power analysis	D	Yes	
**Sample**			
Description	E	Yes	
Volume or mass of sample processed	E	Yes	
Microdissection or macrodissection	E	No	
Processing procedure	E	No	
If frozen—how and how quickly?	E	No	
If fixed—with what, how quickly?	E	No	
Sample storage conditions and duration (especially for formalin-fixed, paraffin-embedded samples)	E	Yes	All samples stored at -20°C
**Nucleic acid extraction**			
Procedure and/or instrumentation	E	Yes	
Storage conditions: temperature, concentration, duration, buffer	E	Yes	In accordance with ISO 21571: 2005. Foodstuffs - Methods of analysis for the detection of genetically modified organisms and derived products - Nucleic acid extraction. Geneva: International Organisation for Standardisation
DNA or RNA quantification	E	No	
Quality/integrity, instrument/method, e.g. RNA integrity/R quality index and trace or 3':5'	E	No	
Template structural information	E	Yes	

Template modification (digestion, sonication, preamplification, etc.)	E	Yes	In accordance with ISO 21571: 2005. Foodstuffs - Methods of analysis for the detection of genetically modified organisms and derived products - Nucleic acid extraction. Geneva: International Organisation for Standardisation.
Template treatment (initial heating or chemical denaturation)	E	Yes	
DNA contamination assessment of RNA sample	E	Yes	
Inhibition dilution or spike	E	No	
Details of DNase treatment where performed	E	No	
Manufacturer of reagents used and catalogue number	D	No	
Storage of nucleic acid: temperature, concentration, duration, buffer	E	Yes	
**RT (If necessary)**			
cDNA priming method + concentration	E	No	
One- or 2-step protocol	E	No	
Amount of RNA used per reaction	E	No	
Detailed reaction components and conditions	E	No	
RT efficiency	D	No	
Estimated copies measured with and without addition of RT	D	No	
Manufacturer of reagents used and catalogue number	D	No	
Reaction volume (for 2-step RT reaction)	D	No	
Storage of cDNA: temperature, concentration, duration, buffer	D	No	
**dPCR target information**			
Sequence accession number	E	Yes	K00821 (*le*1); others are part of patents for each GM-event.
Amplicon location	D	No	
Amplicon length	E	Yes	
In silico specificity screen (BLAST, others)	E	Yes	
Pseudogenes, retropseudogenes or other	D	No	

homologues?			
Sequence alignment	D	No	
Secondary structure analysis of amplicon and GC content	D	No	
Location of each primer by exon or intron (if applicable)	E	No	
Where appropriate, which splice variants are targeted?	E	No	
**dPCR oligonucleotides**			
Primer sequences and/or amplicon context sequence	E	Yes	
RTPrimerDB (real-time PCR primer and probe database) identification number	D	No	
Probe sequences	D	Yes	
Location and identity of any modifications	E	Yes	Modifications of the fluorophores (FAM, HEX) and quenchers (BHQ1)
Manufacturer of oligonucleotides	D	Yes	Eurofins MWG Operon and Integrated DNA Technologies
Purification method	D	Yes	Desalting (HPSF®)
**dPCR protocol**			
Complete reaction conditions	E	Yes	
Reaction volume and amount of RNA/cDNA/DNA	E	Yes	
Primer, (probe), Mg^{2+} and dNTP concentrations	E	Yes	Applicable for primers and probes, other chemicals were part of ddPCR™ Supermix for Probes (No dUTP) (concentrations not disclosed by the manufacturer)
Polymerase identity and concentration	E	No	Not disclosed by the manufacturer
Buffer/kit catalogue no. and manufacturer	E	Yes	#186-3024, Bio-Rad (Hercules, CA, USA)
Exact chemical constitution of the buffer	D	No	Not disclosed by the manufacturer
Additives (SYBR green I, DMSO, others)	E	No	Not disclosed by the manufacturer
Plates/ tubes Catalogue No and manufacturer	D	Yes	#0030128605, Eppendorf (Hamburg, Germany)

Complete thermocycling parameters	E	Yes	
Reaction setup	D	Yes	
Gravimetric or volumetric dilutions (manual/ robotic)	D	Yes	Volumetric manual dilutions
Total PCR reaction volume prepared	D	Yes	10% larger volume was prepared
Partition number	E	Yes	Data available upon request
Individual partition volume	E	Yes	
Total volume of the partitions measured (effective reaction size)	E	Yes	Data available upon request
Partition volume variance/SD	D	No	
Comprehensive details and appropriate use of controls	E	Yes	
Manufacturer of dPCR instrument	E	Yes	
**dPCR validation**			
Optimization data for the assay	D	No	
Specificity (when measuring rare mutations, pathogen sequences)	E	Yes	*In-silico* specificity was checked. Since EURL validated assays were used, specificity for each individual target was not re-tested
Limit of detection of calibration control	D	No	
If multiplexing, comparison with singleplex assays	E	Yes	
**Data analysis**			
Mean copies per partition (λ or equivalent)	E	Yes	Data available upon request
dPCR analysis programme (source, version)	E	Yes	Quantasoft v. 1.6.6.0320 (Bio-Rad; Hercules, CA, USA)
Outlier identification and disposition	E	Yes	
Results of no-template controls	E	Yes	
Examples of positive(s) and negative experimental results as supplemental data	E	Yes	
Where appropriate, justification of number and choice of reference genes	E	No	
Where appropriate, description of	E	No	

normalization method			
Number and concordance of biological replicates	D	No	
Number and stage (RT or dPCR) of technical replicates	E	Yes	
Repeatability (intra-assay variation)	E	Yes	
Reproducibility (interassay/ user/ laboratory variation)	D	Yes	
Experimental variance or CI	E	Yes	Data available upon request
Statistical methods used for analysis	E	Yes	No specific statistical methods were needed (data were analysed by Poisson statistics in Microsoft Excel spreadsheet)
Data submission using RDML (real-time PCR data mark-up language)	D	No	

[a]E, essential; [b]D, desirable

Table S23. Compliance with minimum performance parameters for methods acceptance according to ref. 26[a]

MPP	Definition	AAV	Value reported	Comment	Compliant? (Y/N)
Applicability	Description of the analytes, species, matrices and target concentrations to which the module can be applied, and the type of study/monitoring effort for which the module, judged from its performance, is suited.	An applicability statement shall provide information on the scope of the module and include reference to data documenting the performance of the module. The description should also include warnings to known interferences by other analytes, or inapplicability to certain matrices and situations.	Description of scope, data documenting performance and warnings	See introduction, results and first part of the discussion in the main paper.	Y
Specificity	Property of a method to respond exclusively to the characteristic or analyte of interest.	The module should only produce a positive test result with the target sequence for which the module was developed. This should be demonstrated by *in silico* similarity searches against available and appropriate databases (e.g. EMBL, GenBank, DDBJ, Patent, etc.) and experimentally with suitable samples with and without the presence of the target sequence. *In silico*, it must be demonstrated that the target sequence is unique, and any similarities that theoretically can result in false positives with the analytical module must be reported, and its potential consequences must be briefly discussed. Experimentally, …, it must be demonstrated that no false positives are produced with the samples that do not contain the target sequence, and that in phase *i* (development) at least 95% and in phase *ii* (validation) all samples containing the target sequence yields a positive test result. These tests should be	*In silico* results relating to multiplexing of the primers and probes are reported in the main paper. These indicate that the modules are specific. Experimentally, all samples containing target sequence yielded positive results. The experiments were carried out with sufficient quantity of target DNA.	The specificity of line (event) specific combinations of primers and probes has been verified *in silico* and experimentally in connection with their validation for regulatory compliance by the EURL-GMFF and ENGL (see references in Table 1 of the main paper).	Y

		conducted with approximately 4,000 copies or 10 ng of non-target DNA and with between 100 and 1000 copies or between 0.25 and 2.5 ng of target DNA, unless a more relaxed or strict AAV is justified.			
False positive rate (type I error rate)	The probability *α* of making a type I error (scoring a false positive).<br>Note: In digital PCR two types of false positive results can occur: 1) individual false positive partitions, and 2) a false conclusion about a positive test result for the whole sample. The latter occurs if the rate of (false) positive partitions exceeds the validated false positive rate of the module.	In the case of digital PCR, α ≤0.2% for individual partitions and α ≤5% for the whole sample at all concentrations. Note: A minimum of 20 diverse samples must be tested representing closely related taxa, representative co-occurring impurities and with molecular make-up challenging the exclusivity of the module (e.g., differing only at a few nucleotide positions).<br>Note: The concentrations to be tested should at least include the lower and upper concentrations at which the module is intended for use, and two other concentration levels within that range.<br>Note: In some cases the positive test result is caused by actual presence of the target ... Some CRMs for GMO detection is an example. These CRMs are certified for the presence and quantity of a particular item (e.g., GMO A), but not for the presence or absence of other items (e.g., GMO B). Prudence in the interpretation of unexpected positive results is therefore required.	No false positives observed, for the whole samples, for individual partitions there were up to two false negative partitions detected, which is under the threshold (reactions where there are three or more positive partitions are regarded as positive)	The specificity of line (event) specific combinations of primers and probes has been verified *in silico* and experimentally in connection with their validation for regulatory compliance by the EURL-GMFF and ENGL (see references in Table 1 of the main paper).	Y
False negative rate (type II error rate)	The probability *b* of making a type II error (scoring a false negative).<br>Note: In digital PCR two types of false negative results can occur: 1) individual false negative partitions, and 2) a	In the case of digital PCR, β≤0.2% for individual partitions and β≤5% for the whole sample at all target concentrations ≥ desired LOD.<br>Note: A minimum of 20 diverse samples must be tested representing the largest available diversity of target containing	No false negatives for the whole samples.		Y

	false conclusion about a negative test result for the whole sample. The latter occurs if the rate of (false) negative partitions exceeds the validated false negative rate of the module.	strains or taxa, to challenge the inclusivity of the module.<br>Note: The concentrations to be tested should at least include the lower and upper concentrations at which the module is intended for use, and two other concentration levels within that range.			
Limit of detection (LOD)	The limit of detection is the lowest amount or concentration of analyte in a sample, which can be reliably detected, but not necessarily quantified.	The LOD is the lowest concentration at which the ***probability of detection (POD)*** ≥ 95%, i.e. the lowest concentration yielding a ***false negative rate*** ≤5%.<br>Note: For GMO the AAV is invariant for individual (e.g., a transgene or taxon specific) and combined (transgene + taxon specific) modules.<br>Note: Theoretically the absolute (copy number based) LOD of a PCR module is 5-10 target copies. The absolute LOD of a PCR module should therefore be ≤25 target copies, unless a more relaxed or strict AAV is justified.	LOD <10 copies	See Table 3 and supplementary Tables S3-S6 and S8.	Y
Lower limit of quantification (LOQ)	The lower limit of quantification is the lowest amount or concentration of analyte in a sample that can be reliably quantified with an acceptable level of ***accuracy***.	The LOQ should be = the lowest amount or concentration included in the ***dynamic range***.<br>The LOQ should be assessed experimentally. Estimates of LOQ should be obtained on a sufficient number of test results. For a full validation this should be at least 15, by analogy with the requirement set for the assessment of RSDr. This allows estimating the LOQ in conjunction with the assessment of RSDr.	LOQ <40 copies	See Table 3 and supplementary Tables S3-S6 and S8	Y
Dynamic range	The range of concentrations over which a quantitative module performs in a linear manner with an acceptable	The dynamic range should cover the full concentration range that the module is intended for. This can be expressed as a relative concentration (% relative to a	At least 40 – 2200 copies for the transgene targets (*en bloc*) and 40 – 22000 copies for the *le*1 endogene.	See Figure 1 and Table 3 of the main paper.	Y

	level of ***accuracy***.	specified unit) or absolute concentration (copy number range).<br>Note: Relative concentration = e.g., 0.01% - 10% for GMOs. Absolute concentration = e.g., 10^2 – 10^7 copies per test.<br>Note: For digital PCR platforms the maximum input of template DNA per PCR is often far more limited than for quantitative real-time PCR modules. For digital PCR the linear response is demonstrated by testing of at least 5 concentration levels evenly distributed over at least the full concentration range that the module is intended for and with a sufficiently high number of repetitions (PCRs) to enable the estimation of ***precision***. Independently of the number of partitions in the digital PCR, the test result is considered positive if the number of partitions yielding a positive test response (target detected) exceeds the false positive rate. Moreover, when the majority of partitions yield positive test responses, the number of partitions yielding a negative test response (target not detected) must exceed the false negative rate to be able to calculate a target concentration (estimate).			
**Accuracy (trueness and precision)**	Accuracy is the combined trueness and precision. See figure to the right. Accuracy is decreasing from top left to bottom and right. This is due to decreasing trueness (top to bottom) and decreasing precision (left to right).	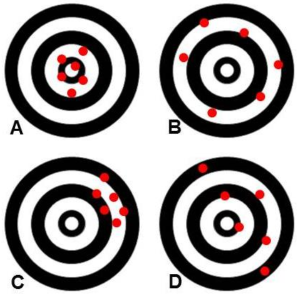	See trueness and precision, respectively		Y
Trueness	The closeness of agreement	The trueness should be within ±25% of the	Trueness within ±25% of the	See Figure 1, Tables 2 and	Y

	between the average value obtained from a large series of test results and an accepted reference value. The measure of trueness is usually expressed in terms of bias. Trueness can therefore be described as the accuracy of the mean.	accepted reference value over the whole ***dynamic range*** of the module.	accepted reference value over the whole ***dynamic range***.	3 and supplementary tables S3-S6 and S8	
Precision (intra and inter laboratory)	The closeness of agreement between independent test results obtained under repeatability (intra laboratory) and reproducibility (inter laboratory) conditions. Repeatability conditions are conditions where test results are obtained with the same module, on identical test items, in the same laboratory, by the same operator, using the same equipment within short intervals of time. Reproducibility conditions are conditions where test results are obtained with the same module, on identical test items, in different laboratories, with different operators, using different equipment. Reproducibility standard deviation describes the inter-laboratory variation.	The relative repeatability standard deviation RSD_r should be ≤25% for individual (e.g., transgene or taxon specific) as well as combined (e.g. transgene + taxon specific) PCR modules over the whole dynamic range. The relative repeatability standard deviation RSD_R ≤35% over the whole dynamic range for individual (e.g. transgene or taxon specific) as well as combined (e.g., transgene + taxon specific) PCR modules), except at concentrations <0.2% or <250 copies where RSD_R ≤50% is acceptable.	RSD_r ≤25% over the whole dynamic range. RSD_R ≤35% over the whole dynamic range when tested.	See Table 3 and supplementary tables S3-S6 and S8	Y
Robustness	The robustness of a module is a measure of its capacity to remain unaffected by small,	The module should provide the expected results when small deviations are introduced from the experimental	Three deviations tested, and with exception for one experiment with the 11-plex	The deviations and results are described in the main paper.	Y (with one minor exception)

	but deliberate deviations from the experimental conditions described in the procedure.	conditions described in the procedure.<br>Note: Examples of small deviations could be ± 50% enzyme concentration, incubation temperature ±2 °C, centrifugation at ±50% time or ±25% × *g*.<br>Note: one measurement/test per deviation is required.	they all yielded acceptable results for all concentrations within the ***dynamic range***.		

[a] Only MPPs applicable to digital droplet and multiplex PCRs are included in the table.

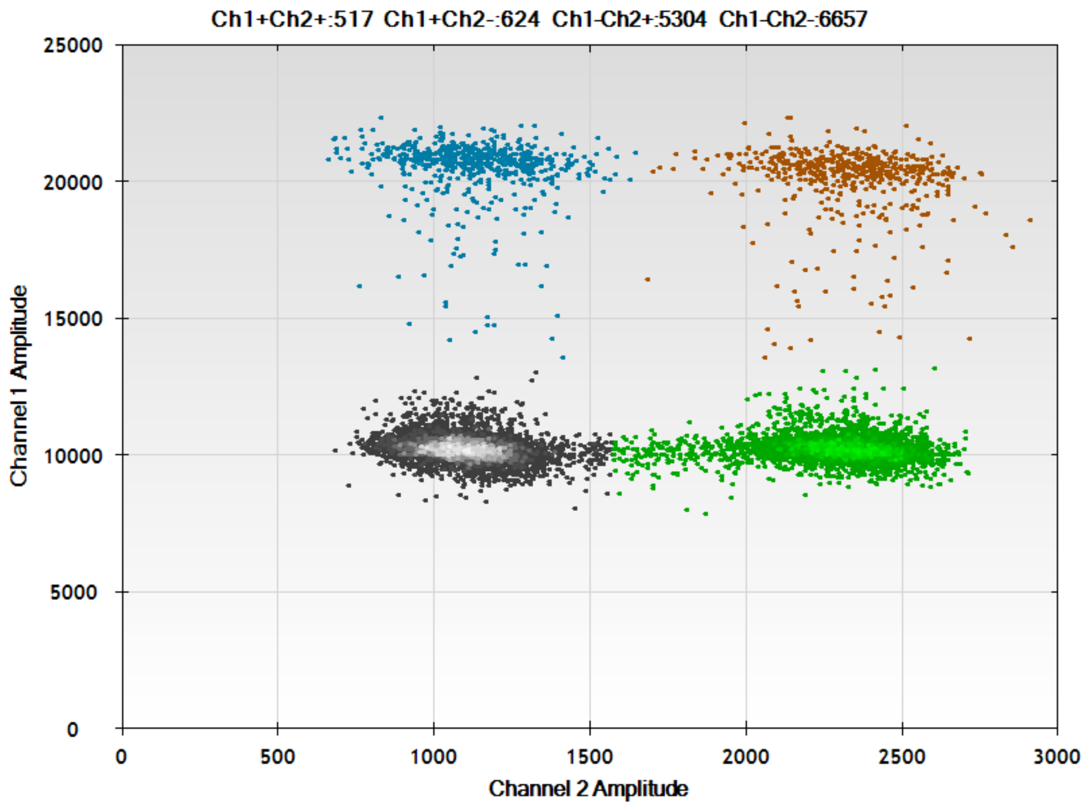

Figure S1. Example of positive experimental results. Fluorescence of droplets in a positive sample (6-plex assay). Droplets in black are negative, droplets in blue are positive for GM-events, droplets in green are positive for endogene (Le1), and those in brown are double positive (positive both for GM-events and Le1).

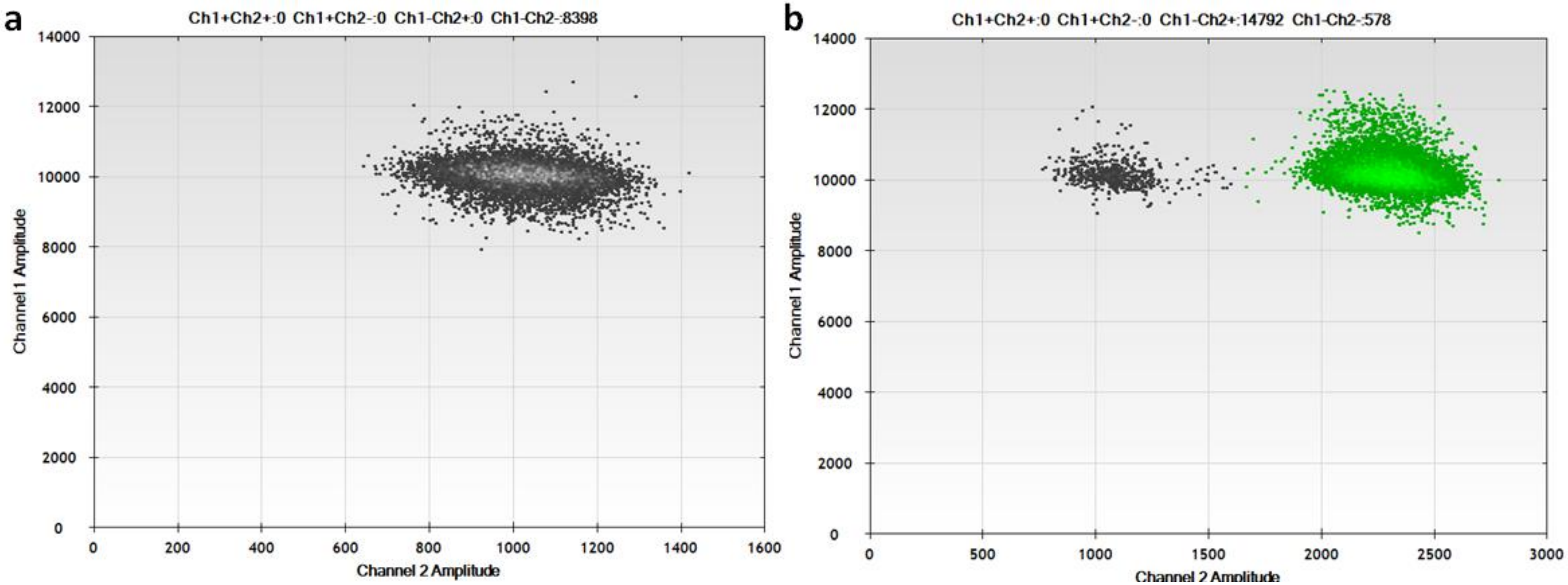

Figure S2. Example of negative experimental results. (a) fluorescence of droplets (black) in no-template control (NTC), (b) fluorescence of negative droplets (black) and only le1 positive droplets (GM negative sample).
